# Gut microbiome differences and disease risk in colorectal cancer relatives and healthy individuals

**DOI:** 10.3389/fcimb.2025.1573216

**Published:** 2025-03-24

**Authors:** Huifen Wang, Weiwei Zhu, Jun Lei, Zhibo Liu, Yudie Cai, Shuaifeng Wang, Ang Li

**Affiliations:** ^1^ Gene Hospital of Henan Province, The First Affiliated Hospital of Zhengzhou University, Zhengzhou, China; ^2^ Department of Infectious Diseases, The First Affiliated Hospital of Zhengzhou University, Zhengzhou, China; ^3^ Department of Gastrointestinal Surgery, The First Affiliated Hospital of Zhengzhou University, Zhengzhou, China; ^4^ State Key Laboratory of Antiviral Drugs, Zhengzhou University, Zhengzhou, China; ^5^ Academy of Medical Sciences, Tianjian Laboratory of Advanced Biomedical Sciences, Zhengzhou University, Zhengzhou, China

**Keywords:** colorectal cancer, family history, gut microbiome, random forest, POD

## Abstract

Given the heightened focus on high-risk populations, this study aimed to provide insights into early susceptibility and preventive strategies for colorectal cancer (CRC) by focusing on high-risk populations. In this research, fecal samples from 1,647 individuals across three discovery cohorts and nine external validation cohorts were sequenced using whole-genome metagenomic sequencing. A prediction model based on random forest was constructed using the nine external cohorts and independently validated with the three discovery cohorts. A disease probability (POD) model based on microbial biomarkers was developed to assess CRC risk. We found that the gut microbiome composition of CRC relatives differed from that of controls, with enrichment of species such as *Fusobacterium* and *Bacteroides* and a reduction in beneficial genera like *Coprococcus* and *Roseburia*. Additionally, dietary red meat intake emerged as a risk factor. The POD model indicated an elevated risk of CRC in unaffected relatives. The findings suggest that the POD for CRC may be increased in unaffected relatives or individuals living in shared environments, although this difference did not reach statistical significance. Our study introduces a novel framework for assessing the risk of colorectal cancer in ostensibly healthy individuals.

## Introduction

Human gut microbiome is a complex microorganism population that possesses 10^14^ microorganisms and is called the second genome of humans (Zhu et al., 2010; Thursby and Juge, 2017). Starting in 1975 for the first research about colorectal cancer (CRC) carcinogenesis in germ-free rats (Reddy et al., 1974), numerous evidence confirmed CRC-associated intestinal microbiome alteration and host–microbiome interaction in CRC patients (Wang et al., 2012; Sheng et al., 2019; Yachida et al., 2019). Multi-cohorts revealed the alteration of gut microbiome in CRC patients and uncovered some distinct increased species across the meta-analysis (Dai et al., 2018; Wirbel et al., 2019). Gavage from CRC patients’ fecal samples to germ-free mice promoted tumorigenesis, indicating that gut microbiome can promote CRC tumorigenesis (Wong et al., 2017). A study has found that *Fusobacterium nucleatum* (*F. nucleatum*) is enriched in colorectal cancer (CRC) tumor tissues with KRAS p.G12D mutations and can promote the occurrence of colorectal tumors in Villin-Cre/KrasG12D^+/−^ mice. This indicates that the carcinogenic effect of *F. nucleatum* is dependent on somatic genetics and the intestinal microbiota ecology. More interestingly, mice orally administered with *Parabacteroides distasonis* (*P. distasonis*) showed a reduction in *F. nucleatum*-dependent CRC progression. This suggests that *P. distasonis* competes with *F. nucleatum* in CRC. Consequently, personalized modulation of the gut microbiota may provide a more targeted therapeutic strategy for CRC treatment (Feng et al., 2015).

A CRC family history (FH) is a recognized risk factor for both hereditary and sporadic colorectal cancer (CRC), owing to shared cohabitation exposures and heritability across family members (Fuchs et al., 1994; Smith et al., 2002). Unaffected relatives of CRC refer to individuals who are family members of colorectal cancer (CRC) patients but do not themselves have a diagnosis of CRC. Up to now, the largest research which included 2 million individuals reported the familial relative risk (FFR) for unaffected persons with at least one affected first-degree relative (FDR) of 2.05, (Taylor et al., 2010) which was consistent with other reports estimating from 1.55 to 2.80. This risk was much higher than individuals with no FDR family history of 0·89 (Taylor et al., 2010; Lowery et al., 2016).

Individuals with their FDRs and second-degree relatives (SDRs) share a genetic background and have more similar lifestyles compared with the background population. Cohabitation and genetic factors both participate in shaping the gut ecosystem, and the former is a major contributor (Gacesa et al., 2022). Gut microbiome transmits among kinship in multigeneration households (Dill-McFarland et al., 2019; Valles-Colomer et al., 2022). A study in 2011 interpreted asymptomatic intestinal dysbiosis in the unaffected relatives of Crohn’s disease (CD) patients (Joossens et al., 2011). Using quantitative PCR targeting 16S ribosomal RNA, altered species of healthy relatives of CD patients were discovered, including a significant decreased abundance of *Faecalibacterium prausnitzii* and *Clostridia cluster IV* compared with controls, which were accordant to the alteration of CD patients (Hedin et al., 2014).

In this study, we submitted a total of 1,647 samples for whole-genome metagenomic sequencing. We investigated the composition of fecal microbiota, functional changes in microbial communities, and the identification of microbial biomarkers. We hypothesize that the CRC, CRC.Relative, and Control groups may exhibit distinct intestinal microbiota profiles, which could elucidate the differences in gut microbiota between the CRC.Relative group and both the CRC and Control groups. This exploration may provide insights into the pathogenesis of CRC across different populations and assess whether CRC.Relative serves as a potential risk factor for the development of CRC.

## Materials and methods

### Participant recruitment and study design

Participants were recruited from the First Affiliated Hospital of Zhengzhou University (January 2018–April 2023) and classified into two cohorts. The 2023zhengzhou cohort included CRC patients (ward.CRC, n = 10), their healthy first-degree relatives (ward.Relative, n = 10), and healthy ward staff without a family history of CRC (ward.Staff, n = 19). The 2018 cohort comprised healthy individuals with a family history of colorectal cancer (HC.Relative, n = 38) and healthy controls without a family history of colorectal cancer (HC.Control, n = 49). Exclusion criteria included participants under 18 and those with recent antibiotic or probiotic use (within 3 months), irritable bowel syndrome (IBS), hypertension, diabetes, gastrointestinal diseases, or other tumors.

For model training, nine external cohorts from eight studies (n = 1,489) were included. The inclusion criteria comprised study cohorts from different countries and regions, sourced from recently published and widely recognized studies, all of which underwent whole-genome sequencing. The model testing phase involved the Zhengzhou2022 cohort, which consisted of 14 CRC patients and 18 healthy controls from the hospital’s physical examination center. In total, three discovery cohorts (Zhengzhou2018, Zhengzhou2023, Zhengzhou2022) and nine external cohorts were included. Data on environmental exposures, including diet, smoking, alcohol use, physical activity, illnesses, medication, and family health history, were collected via structured questionnaires.

### Fecal sample collection and handling

Fecal samples were collected from participants according to a standardized protocol to preserve sample integrity for whole-genome sequencing. Approximately 5 g of fecal matter was collected at the time of natural defecation using sterile tools and immediately transferred to pre-labeled, sterile sampling tubes. The samples were temporarily stored at 4°C and transported to the laboratory under cold chain conditions using dry ice. Upon receipt, sample information was verified, and the tubes were stored at −80°C until further processing.

### Genomic DNA extraction and library construction

Genomic DNA was extracted from 87 fecal samples using the MagPure Stool DNA KF Kit B (Magen, Guangzhou, China) following the manufacturer’s instructions. DNA quantification was performed using a Qubit Fluorometer and the Qubit dsDNA BR Assay Kit (Invitrogen, USA), with validation through electrophoresis on a 1% agarose gel. For library construction, 1 μg of genomic DNA was randomly fragmented using a Covaris system, and the resulting fragments were selected by magnetic beads, targeting an average size of 200–400 bp. These fragments underwent end repair, 3′ adenylation, adapter ligation, and PCR amplification, followed by purification. The double-stranded PCR products were heat-denatured and circularized using a splint oligo sequence. The final library was constructed from single-strand circular DNA (ssCir DNA), and then qualified and sequenced on the MGI SEQ 2000 platform (BGI Shenzhen, China).

### Sequencing data analysis

Taxonomic annotation was performed using MetaPhlAn2, which quantified microbial taxa including bacteria, archaea, eukaryotes, and viruses with default settings. Taxon-specific functional profiles were generated using HUMAnN2 (the HMP Unified Metabolic Analysis Network 2). Microbiome diversity was assessed using several alpha diversity indices: Shannon, Simpson, Gini, and Obs, calculated through the “vegan” package in R. For microbiome feature comparison, a two-tailed Wilcoxon rank-sum test was applied. A P-value less than 0.05 was considered statistically significant. Taxa were first assigned a pseudo-count of 1e−05, followed by log10 transformation for normalization. The normalized abundance of gut microbiome features was then used for comparison.

The comparison of alpha diversity between groups was performed using a Student’s t-test. Between-individual differences were evaluated using principal coordinate analysis (PCoA) based on Spearman distance, implemented in the “ade4” package.

### External dataset and probability of disease model construction

To calculate the Probability of Disease (POD) indices for the HC.Relative, HC.Control, Ward.CRC, Ward.Relative, and Ward.Staff groups, we utilized nine cohorts from eight published studies as external datasets. The POD index, initially introduced in a previous study, is defined as the ratio of an individual’s likelihood of having colorectal cancer (CRC) to their likelihood of being healthy, derived from randomly constructed decision trees.

We identified overlapping species between statistically significant species from the nine external cohorts and those detected in the Zhengzhou cohorts, considering these as potential microbial biomarkers. The most critical biomarkers from these overlapping species were selected based on their feature importance score (mean decrease accuracy) using a random forest model. The POD model, constructed from the nine external cohorts, was applied to individuals from the Zhengzhou cohort to estimate their probability of developing CRC. The receiver operating characteristic (ROC) curve, generated using the “pROC” package, was employed to assess model performance, and the area under the ROC curve (AUC) was used to quantify their effectiveness.

### Statistical analysis

All of the statistical analyses were used by R version 4.2.0. Quantitative variables were compared a using two-tailed Student’s t-test, which was displayed as meanSD. Categorical variables were compared through Fisher’s exact test. Statistical significance was considered as P-value < 0.05.

### Ethics statement

All methods were carried out in accordance with relevant guidelines and regulations. Human participants’ involvement was approved by Ethics Review Committee of Scientific Research Projects in The First Affiliated Hospital of Zhengzhou University. The ethics approval number is 2021-KY-0934. All of the participants provided informed consent for collecting feces samples.

## Results

### Study design and flow diagram

**Figure 1:**
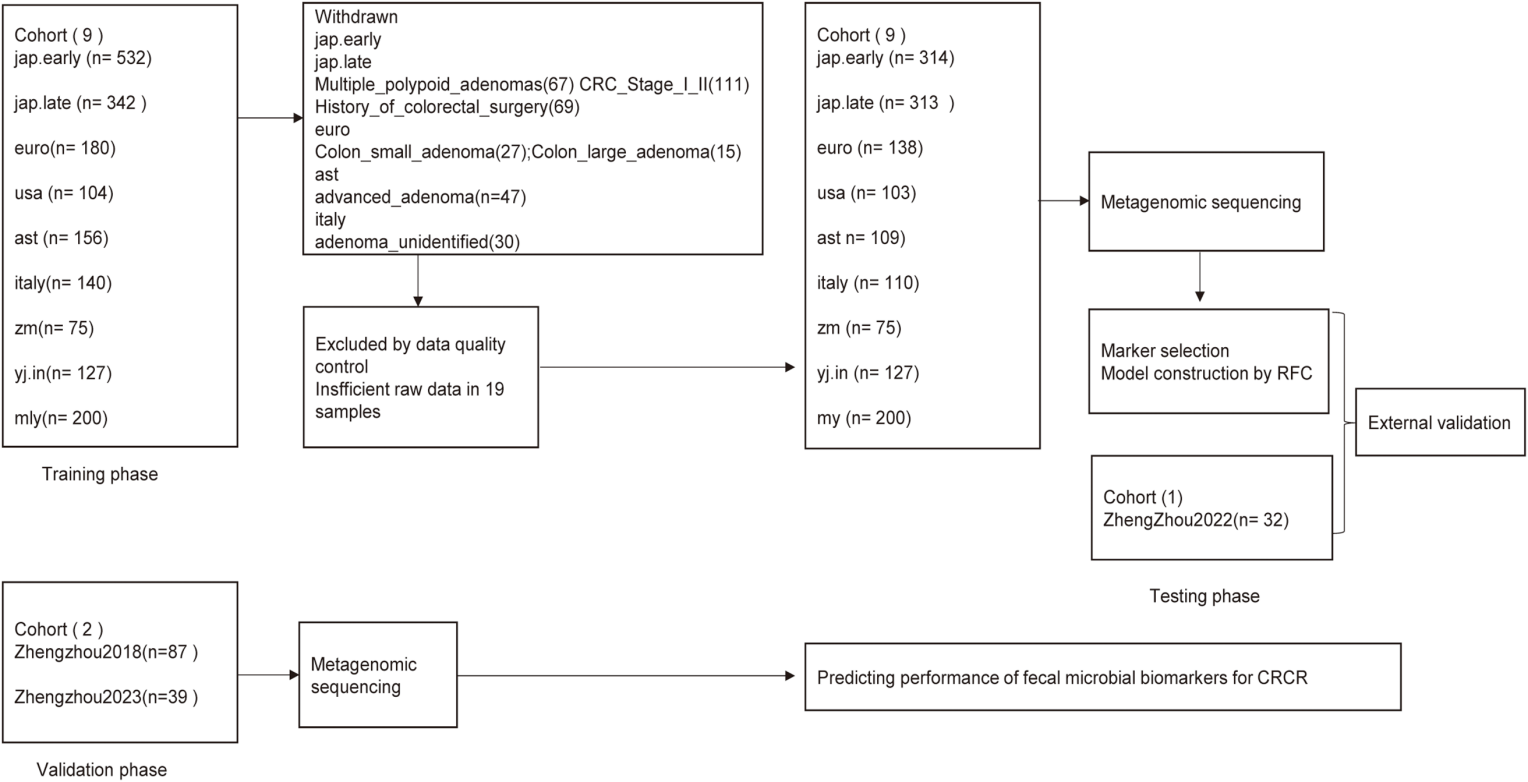
Study design and schematic diagram. This study enrolled a total of 1,678 eligible cases. Fecal microbiota was analyzed using metagenomic sequencing. Nine published cohorts of colorectal cancer (CRC) patients and age-matched healthy controls were randomly selected for the training phase, whereas the Zhengzhou2022 cohort was used as the internal testing phase to identify gut microbial communities and microbial markers. The identified microbial markers were further validated independently in two additional Zhengzhou cohorts: Zhengzhou2018 with 49 HC.Controls and 38 HC.Relatives, and Zhengzhou2023 comprising 10 Ward.CRC, 10 Ward.Relatives, and 19 Ward.Staff during the external validation phase. CRC, colorectal cancer; Relative, relative of colorectal cancer patients; Control, age-matched healthy control group; RFC, random forest classifier.

Following a rigorous recruitment process, a total of nine established cohorts and three discovery cohorts, comprising 1,647 eligible cases, were enrolled (**Figure 1**). Metagenomic sequencing (n = 1,647) was conducted to analyze the fecal microbiota associated with all medical records. During the training phase, data from nine published cohorts comprising 1,489 samples from diverse geographic regions were utilized. A random forest model was employed to identify microbial biomarkers and develop a classifier to distinguish colorectal cancer (CRC) patients from age-matched healthy controls.

For the testing phase, 14 CRC cases and 18 control cases from the Zhengzhou2022 cohort were utilized to validate the classifier’s performance in distinguishing CRC from healthy individuals. Furthermore, to assess the classifier’s ability to differentiate between healthy individuals with familial ties to CRC patients and those without such ties, two independent external validation cohorts were included: The Zhengzhou2018 cohort, consisting of 49 HC.Controls and 38 HC.Relatives, and the Zhengzhou2023 cohort, comprising 10 Ward.CRC cases, 10 Ward.Relatives, and 19 Ward.Staff.

### Participants’ characteristics

In the zhengzhou2018 cohort, there were 24 male CRC relatives and 14 female CRC relatives. The baseline characteristics of the HC.Control group (n = 49) and the HC.Relative group (n = 38) were exhibited (**Table 1**). No significant difference was found between gender, age, and anthropometrics addition to lifestyles. At the dietary level, there was no significant difference between the two groups in the intake of grains and probiotics. However, a notable difference was found in the average weekly consumption of red meat between the two groups. There were no significant differences between the two groups in terms of white meat intake. Laboratory tests showed no statistically significant differences in blood indexes, apart from CYFRA211, a tumor marker for lung cancer, which had a higher trend in HC.Relative. Among the 38 participants in the HC.Relative group, 29 participants had a family history of FDR, whereas 9 participants had a family history of SDR.

**Table 1:** Demographics of the participants in the zhengzhou2018 cohort.

Feature	HC.Relative (n=38)	HC.Control (n=49)	P value
Gender (n)			
Female	17	22	1.000
Male	21	27	
BMI (kg/m^2^) [mean (SD)]	24.027 (3.595)	25.481 3.777)	0.077
Age (year) [mean (SD)]	44.079 (11.917)	46.082 11.733)	0.436
Waist (cm) [mean (SD)]	83.013 (11.974)	86.908 11.216)	0.126
Hip (cm) [mean (SD)]	97.2 (6.326)	99.293 12.486)	0.329
Laboratory tests [mean (SD)]			
RBC (10^12^/L)	4.718 (0.431)	4.777 (0.465)	0.540
WBC (10^9^/L)	6.096 (1.423)	5.942 (1.725)	0.650
PLT (10^9^/L)	228.974 (41.86)	228.51 (64.34)	0.968
ALT (U/L)	23.211 (16.064)	26.592 (21.548)	0.404
AST (U/L)	21.684 (9.358)	22.531 (8.198)	0.660
Creatinine (μmol/L)	69.158 (12.614)	69.49 (14.589)	0.910
CA72-4 (U/ml)	4.767 (9.034)	4.022 (6.239)	0.855
CEA (ng/ml)	1.5 (0.964)	1.848 (1.025)	0.167
CYFRA211 (ng/ml)	2.077 (0.699)	1.197 (0.295)	0.015
CA19-9 (U/ml)	13.286 (11.494)	13.893 (9.865)	0.882
CA15-3 (U/ml)	8.758 (3.128)	15.242 (6.349)	0.054
CA125 (U/ml)	17.967 (14.109)	14.837 (10.315)	0.481
NSE (ng/ml)	12.864 (1.938)	11.873 (4.292)	0.592
AFP (ng/ml)	2.868 (1.212)	2.666 (1.671)	0.717
Lifestyles [n(n%)]			
Whole grains			
No	9 (23.68)	11 (22.45)	1.000
Yes	29 (76.32)	38 (77.55)	
Probiotics			
No	18 (47.37)	14 (28.57)	0.116
Yes	20 (52.63)	34 (69.39)	
Red meat >150 g/w			
No	12 (31.60)	32 (65.30)	0.002
Yes	26 (68.40)	17 (34.70)	
White meat >150 g/w			
No	18 (47.40)	29 (59.20)	0.189
Yes	20 (52.60)	20 (40.80)	
Smoking			
No	29 (76.32)	42 (85.71)	0.253
Yes	9 (23.68)	6 (12.24)	
Drinking			
No	27 (71.05)	28 (57.14)	0.487
Yes	11 (28.95)	17 (34.69)	

Continuous variables were compared using Student’s t-test and categorical variables were compared using Fisher’s exact test.

SD, standard deviation; BMI, body mass index; RBC, red blood cell; WBC, white blood cell; PLT, platelet; ALT, alanine aminotransferase; AST, aspartate aminotransferase; CA72-4, cancer antigen 72-4; CEA, carcinoembryonic antigen; CYFRA211, cytokeratin fragment 19; NSE, neuron-specific enolase; AFP, alpha fetoprotein.

In the Zhengzhou2023 cohort, there were 10 CRC cases, 10 first-degree relatives of CRC patients (CRC Relatives), and 19 staff members (Ward.Staff). Baseline characteristics of the Ward.CRC group (n = 10), Ward.Relative group (n = 10), and Ward.Staff group (n = 19) are presented in **Table 2**. Significant differences were observed in age and BMI among the three groups. No significant differences were found in gender, anthropometric measurements, or lifestyle factors such as whole grains, probiotics, smoking, or drinking. Interestingly, No significant differences were observed among the three groups in terms of weekly red meat and white meat intake. A comparison of colonoscopy history within the past 5 years revealed significant differences among the groups. All patients in the Ward.CRC group had undergone colonoscopy, whereas none of their first-degree relatives (Ward.Relatives) had. In contrast, approximately half of the individuals in the Ward.Staff group had undergone colonoscopy during this period.

**Table 2:** Demographics of the participants in the zhengzhou2023 cohort.

Feature	Ward.CRC (n=10)	Ward.Relative (n=10)	Ward.Staff (n=19)	P value
Gender (n)				
Female	5	5	10	1.000
Male	5	5	19	
Age (year) [mean (SD)]	58.9(4.032)	38.5(4.443)	44.26(2.123)	0.001
BMI (kg/m2) [mean (SD)]	22.2(0.987)	22.9(0.674)	25.2(0.852)	0.049
Age (year) [mean (SD)]	44.079(11.917)	46.082(11.733)	46.082(11.733)	0.436
Waist (cm) [mean (SD)]	72.7(1.325)	75.8(6.46)	83.09(10.818)	0.009
Hip (cm) [mean (SD)]	163.6(2.596)	167.5(2.557)	169.276(1.9)	0.223
Weight(kg)[mean(SD)]	60.3(4.417)	64.2(3.073	72.763(3.412)	0.055
Lifestyles [n(n%)]				
Whole grains				
No	3(30.00)	2(20.00)	4(21.05)	0.889
Yes	7(70.00)	8(70.00)	15(78.95)	
Probiotics				
No	6(60.00)	4(40.00)	8(42.11)	0.707
Yes	4(40.00)	6(60.00)	11(57.89)	
Red meat >150 g/w				
No	6(60.00)	5(50.00)	8(42.1)	0.920
Yes	4(40.00)	5(50.00)	11(57.9)	
White meat >150 g/w				
No	6(60.00)	5(50.00)	9(47.4)	1.000
Yes	4(40.00)	5(50.00)	10(52.6)	
Smoking				
No	6(60.00)	8(80.00)	10(52.63)	0.473
Yes	4(40.00)	2(20.00)	9(47.37)	
Drinking				
No	7(70.00)	7(70.00)	11(57.89)	0.755
Yes	3(30.00)	3(30.00)	8(42.11)	
Colonoscopy within 5 years				
No	0(0.00)	10(100.00)	9(47.37)	0.001
Yes	10(100.00)	0(0.00)	10(52.63)	

Continuous variables were analyzed using analysis of variance (ANOVA), whereas categorical variables were assessed using the chi-square test.

### Comparison of gut microbiota diversity in the Zhengzhou cohorts

**Figure 2:**
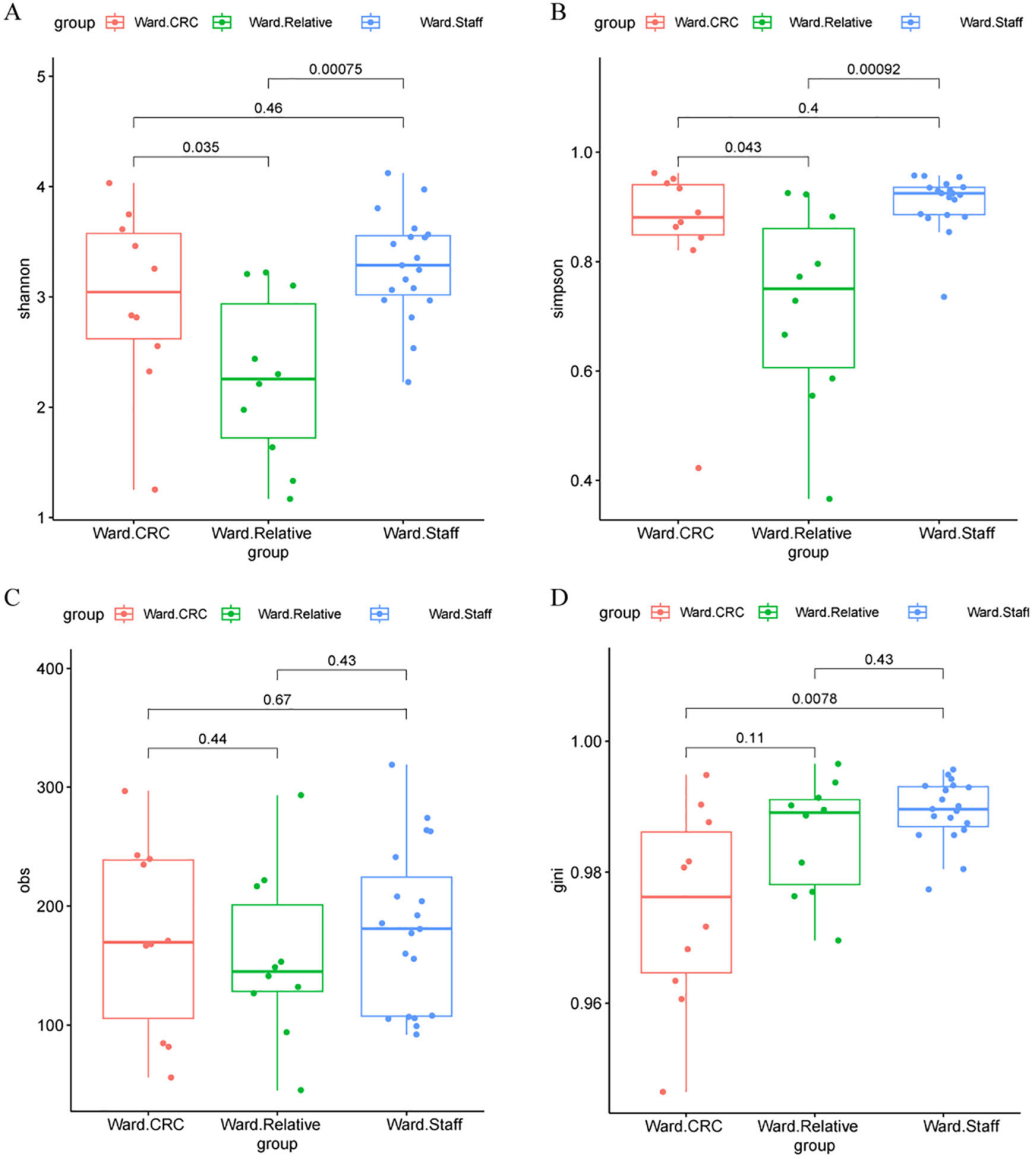
Alpha diversity metrics in the Ward.CRC, Ward.Relative, and Ward.Staff groups. **(A)** Shannon diversity index comparison among Ward’s CRC, relative, and staff groups, with no significant differences observed (Wilcoxon tests, P values for CRC vs. Relative = 0.00075, CRC vs. Staff = 0.035, Relative vs. Staff = 0.46). **(B)** Simpson diversity index comparison, showing significant differences between CRC and relative groups (P = 0.043), but not between other group pairs. **(C)** Observed species (obs) metric comparison, with significant differences between CRC and relative groups (P = 0.44), and between CRC and staff groups (P = 0.67). **(D)** Gini coefficient comparison, showing significant differences between CRC and relative groups (P = 0.0078), and between CRC and staff groups (P = 0.11). These results suggest that certain alpha diversity metrics vary significantly between Ward’s CRC and other groups, potentially indicating a relationship with colorectal cancer status.

Alpha diversity of the gut microbiota was evaluated using several indices, including the Shannon index, Simpson index, Observed species (Obs) index, and Gini index across three Zhengzhou cohorts. Significant differences were found in the Shannon and Simpson indices between the Ward.CRC and Ward.Relative groups, as well as between the Ward.Relative and Ward.Staff groups, indicating variations in microbial diversity. Additionally, a notable difference in the Gini index was observed between the Ward.CRC and Ward.Staff groups, suggesting that microbial evenness differed between these groups. However, no significant differences were detected for the Obs index across these groups. The Ward.Relative group exhibited lower alpha diversity compared with the Ward.Staff group, suggesting that the microbiota of CRC patients’ relatives may have been less diverse (Zhengzhou2023 cohort, **Figures 2A–D**).

**Figure 3:**
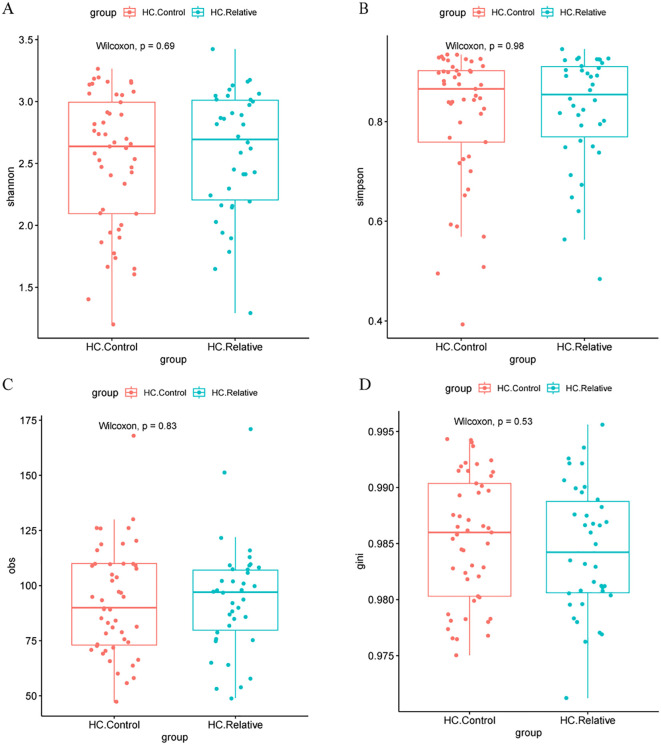
Alpha diversity metrics in HC.Controls and HC.CRC patients**(A)** Comparison of Shannon diversity index between healthy controls (HC.Control) and relative colorectal cancer patients (HC.Relative). No significant difference was observed (Wilcoxon test, P = 0.69). **(B)** Simpson diversity index comparison between the same groups, also showing no significant difference (Wilcoxon test, P = 0.98). **(C)** Observed species (obs) metric comparison, with no significant difference between groups (Wilcoxon test, P = 0.83). **(D)** Gini coefficient comparison, again with no significant difference (Wilcoxon test, P = 0.53). These results suggest no significant differences in alpha diversity metrics between the two groups.

In contrast, no significant differences in alpha diversity indices, including the Shannon, Simpson, Obs, or Gini indices, were found between the HC.Control and HC.Relative groups (Zhengzhou2018 cohort **Figures 3A–D**). This suggests that the gut microbiota diversity of first-degree relatives of colorectal cancer (CRC) patients does not significantly differ from that of healthy individuals without a family history of CRC. Similarly, no notable differences in alpha diversity indices were observed between the zzu.Control and zzu.CRC groups (zhengzhou2022 cohort, **Supplementary Figures S1A–D**).

### Principal coordinate analysis

**Figure 4:**
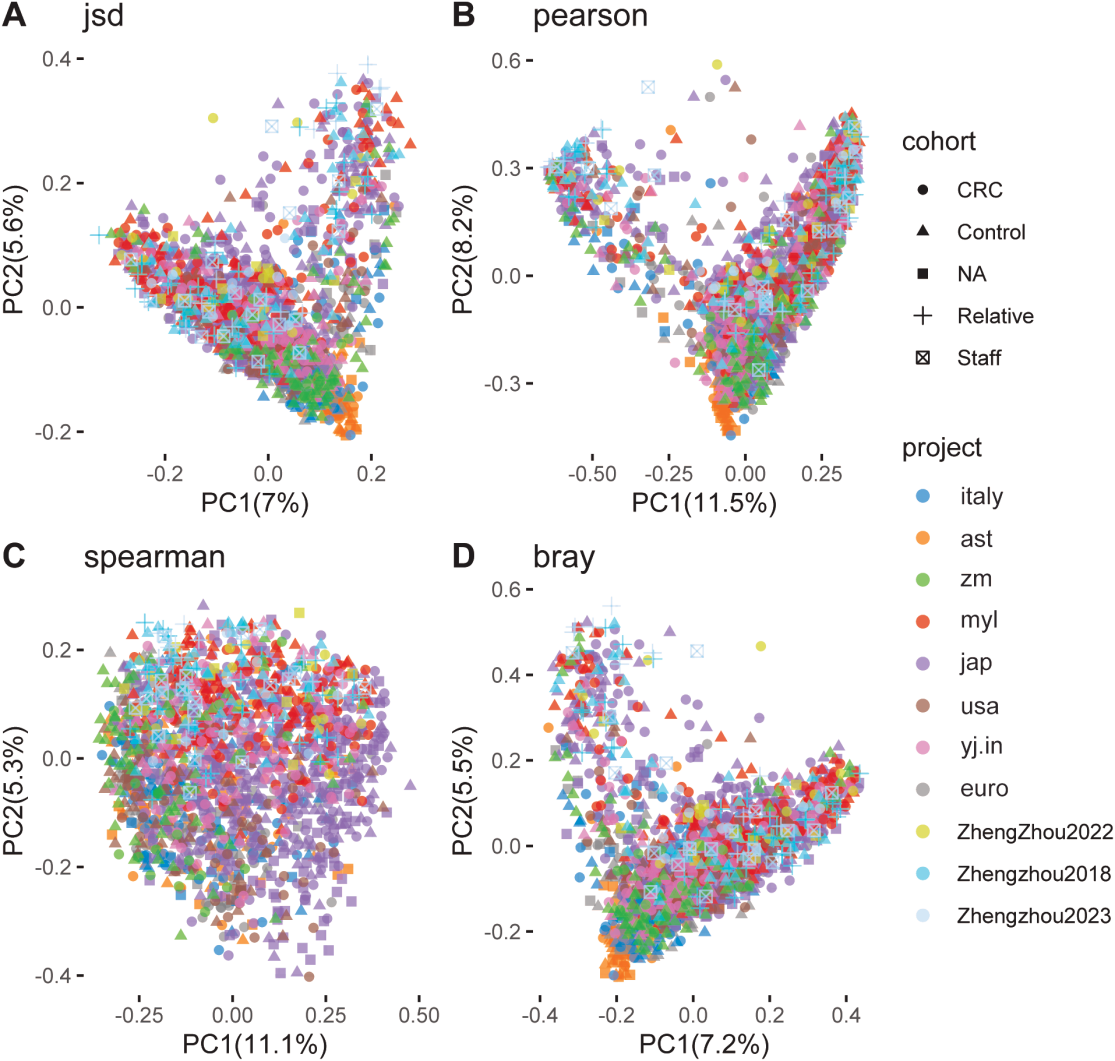
Principal coordinate analysis (PCoA) of gut microbiome communities from various cohorts and projects. Using different distance metrics to evaluate the relationships between colorectal cancer (CRC) patients and control groups. **(A)** PCoA based on Jensen–Shannon divergence, showing a clear separation between CRC and control groups along the first two principal components (PC1 and PC2), which account for 17.5% and 6.8% of the variance, respectively. **(B)** PCoA based on Pearson correlation, with PC1 and PC2 explaining 11.5% and 5.3% of the variance, respectively. **(C)** PCoA based on Spearman correlation, where PC1 and PC2 account for 11.1% and 6.3% of the variance, respectively. **(D)** PCoA based on Bray–Curtis dissimilarity, with PC1 and PC2 explaining 17.2% and 5.5% of the variance, respectively. Each plot includes data from multiple studies, such as Andrew2019, Feng2015, Jakob2019, Myl2021, Shinichi2019, Vogt2016, Yj2015, Zeller2014, Zhengzhou2022, and Zhengzhou2023, color-coded by cohort (CRC, Control, NA, Relative, Staff) and project.

Principal coordinate analysis (PCoA), based on Spearman, Pearson, Jensen–Shannon, and Bray–Curtis distances, was conducted to evaluate the clustering of microbiomes in this study. The analysis included nine established cohorts and three Zhengzhou cohorts. The CRC, Control, NA, Relatives, and Staff groups were clearly separated along the first and second principal components, which explained 7.0% and 5.6%, 11.5% and 8.2%, 11.1% and 5.3%, and 7.2% and 5.5% of the variance, respectively (**Figures 4A–D**).

**Figure 5:**
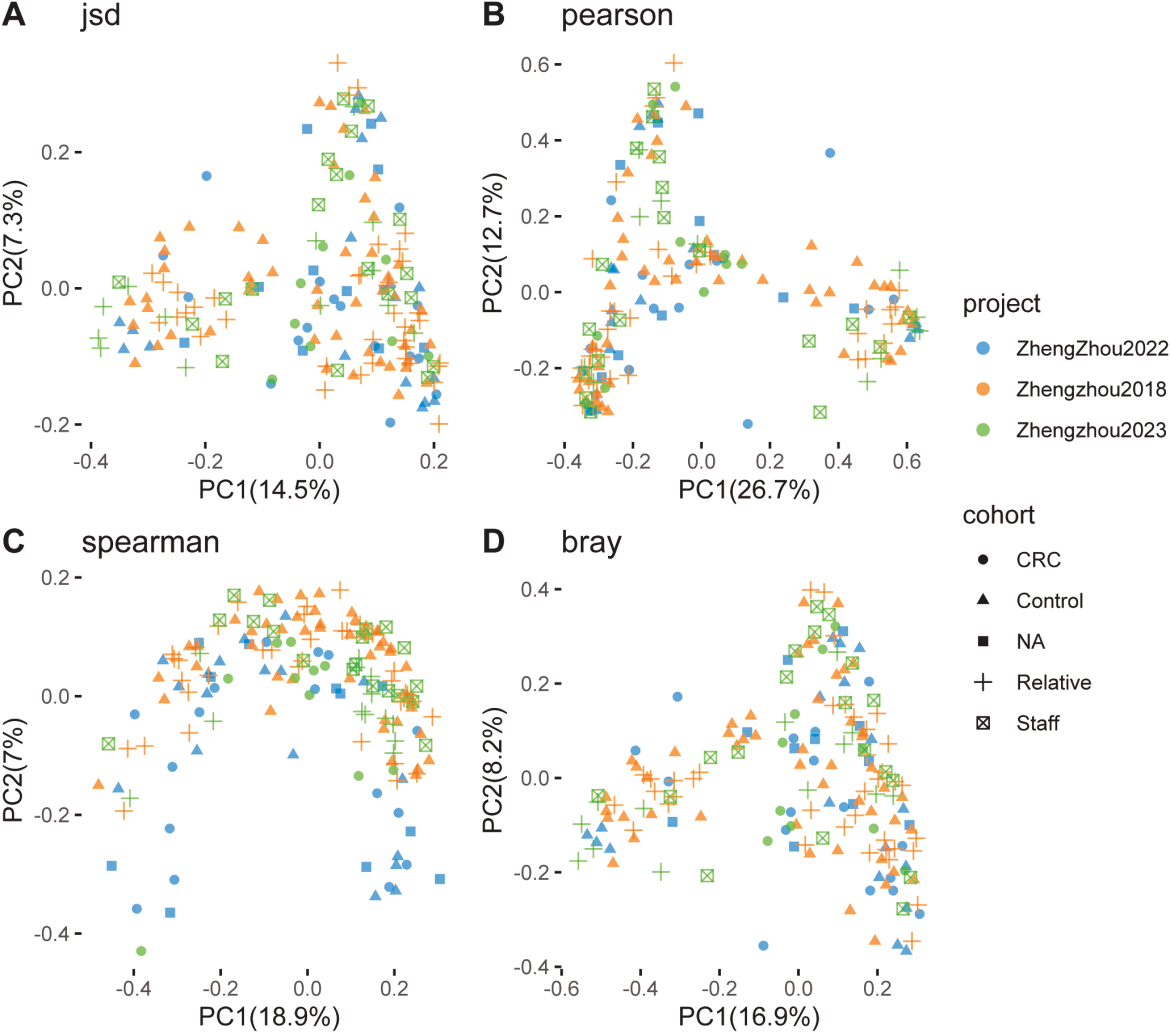
Principal coordinate analysis (PCoA) of gut microbiome communities. Focusing on the separation between colorectal cancer (CRC) patients and control groups using different distance metrics. **(A)** PCoA based on Jensen–Shannon divergence, with the first two principal components (PC1 and PC2) explaining 14.5% and 7.7% of the variance, respectively. **(B)** PCoA based on Pearson correlation, where PC1 and PC2 account for 26.7% and 7.7% of the variance, respectively. **(C)** PCoA based on Spearman correlation, with PC1 and PC2 explaining 9.9% and 6.8% of the variance, respectively. **(D)** PCoA based on Bray–Curtis dissimilarity, with PC1 and PC2 accounting for 16.9% and 10.9% of the variance, respectively. The plots include data from studies such as Zhengzhou2022 and Zhengzhou2023, color-coded by cohort (CRC, Control, NA, Relative, Staff) and project. The clustering patterns reflect the differences in microbiome composition among the various cohorts and projects, suggesting potential associations with CRC status.

Similarly, within the three Zhengzhou cohorts, the CRC, Control, NA, Relatives, and Staff groups were also distinct along the first and second principal components, accounting for 14.5% and 7.3%, 26.7% and 12.7%, 18.9% and 7.0%, and 16.9% and 8.2% of the variance, respectively (**Figures 5A–D**). These results demonstrate a clear differentiation of microbial communities between the various groups within both the established and Zhengzhou cohorts. This indicates that microbial compositions are group-specific and potentially influenced by factors such as disease status (CRC), familial relationships (Relatives), or environmental/lifestyle differences (Staff). Moreover, similar clustering patterns observed in both the established and Zhengzhou cohorts support the robustness of the findings. It suggests that the identified microbial differences are reproducible across independent cohorts with varying characteristics.

### Analysis of gut microbiota composition among different groups across three cohorts

To investigate the differences in microbial composition among various groups, we performed statistical analyses using STAMP software to compare the microbial composition across the three Zhengzhou cohorts.

In zhengzhou2022 cohort, the abundance of *Prevotella* and *Phocaeicola* was decreased in the CRC group compared with the control group. Studies suggest that changes in the abundance of *Phocaeicola* may be linked to various disease states, including colorectal cancer (CRC), inflammatory bowel disease (IBD), and metabolic disorders. For instance, in some CRC patients, the abundance of *Phocaeicola* may be significantly reduced, which could be related to its metabolic characteristics or its potential influence on the tumor microenvironment.

The impact of *Prevotella* on CRC may be dual-faceted, depending on factors such as strain specificity, host genetic background, lifestyle (e.g., dietary patterns), and the overall state of the gut ecosystem. (**Supplementary Figure S2**).

**Figure 6:**
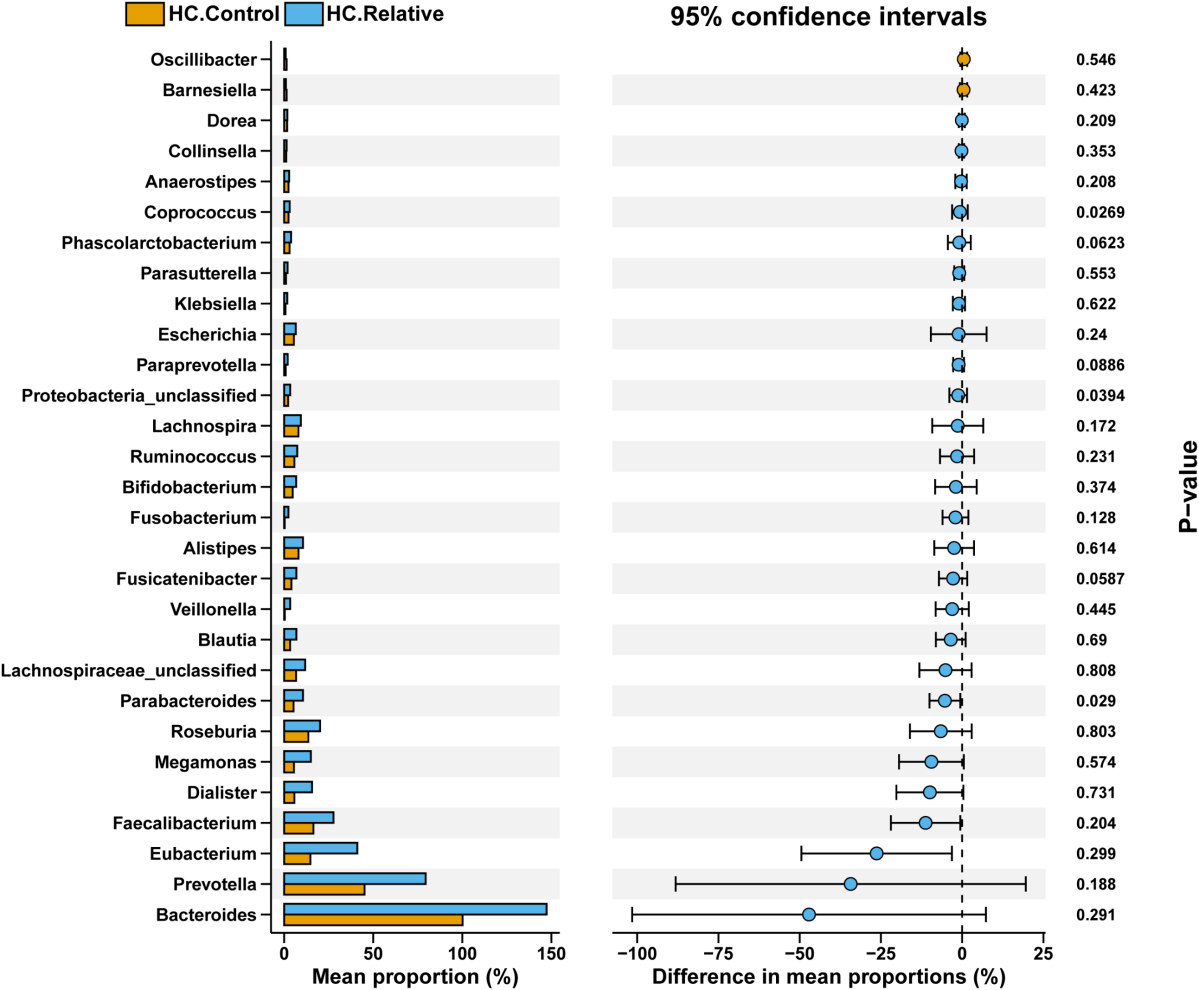
Comparison of gut microbial composition between HC.Control and HC.Relative groups. The bar plot on the left shows the mean proportion (%) of each bacterial genus in the two groups, highlighting dominant taxa such as *Bacteroides*, *Prevotella*, *Faecalibacterium*, and others. The dot plot on the right illustrates the difference in mean proportions (%) between groups, with 95% confidence intervals. Asterisks denote significant differences with P-values labeled. The data suggest distinct microbial profiles between the groups, with notable variations in specific genera that may be relevant to group-specific characteristics.

In the zhengzhou 2018 cohort, significant differences in the abundances of *Parabacteroides*, *Proteobacteria_unclassified*, and *Coprococcus* were observed between the HC.Control and HC.Relative groups (P < 0.05). Research has shown that the overall abundance of *Proteobacteria* tends to increase in some colorectal cancer (CRC) patients. Specifically, *Proteobacteria_unclassified* may contribute to CRC progression by producing endotoxins such as lipopolysaccharides (LPS), disrupting the intestinal barrier, or inducing inflammatory responses. The effects of *Parabacteroides* and *Coprococcus* on gut health appear to be context-dependent, often exhibiting dual roles. Their abundance changes are associated with disease states, gut dysbiosis, and elevated inflammation levels. Notably, *Bacteroides* and *Prevotella* exhibited relatively high abundances in both groups, with significantly higher levels in the HC.Relative group compared with the HC.Control group. Previous studies have reported that *Bacteroides* abundance is generally stable in a healthy gut but may increase in CRC patients. This increase is thought to contribute to CRC development by altering the composition of metabolic byproducts, promoting inflammation, or modifying the gut environment. These findings suggest that the HC.Relative group may exhibit microbiota changes potentially linked to familial CRC risk (**Figure 6**).

In zhengzhou 2023 cohort, a significant reduction in *Prevotella*, *Lachnospira*, *Roseburia*, and *Lachnospiraceae_unclassified* was observed in the Ward.CRC group compared with the Ward.Relative group, whereas *Escherichia* was significantly increased. In a healthy gut, the abundance of *Roseburia* is typically higher, and a decrease in *Roseburia* in colorectal cancer (CRC) patients may result in insufficient butyrate production, which in turn promotes the onset and progression of intestinal pathology. Similarly, a reduction in *Lachnospira* and *Lachnospiraceae_unclassified* may limit short-chain fatty acid (SCFA) production, leading to gut microbiota dysbiosis and inflammation. On the other hand, *Escherichia* abundance is usually elevated in CRC, particularly with an increase in pathogenic strains of *Escherichia coli*, which may serve as an important indicator of CRC development (**Supplementary Figure S3**). Similarly, in the comparison between the Ward.CRC and Ward.Staff groups, the abundance of *Prevotella*, *Lachnospira*, *Escherichia*, and *Bacteroides* exhibited trends similar to those observed between the Ward.CRC and Ward.Relative groups (**Supplementary Figure S4**).

**Figure 7:**
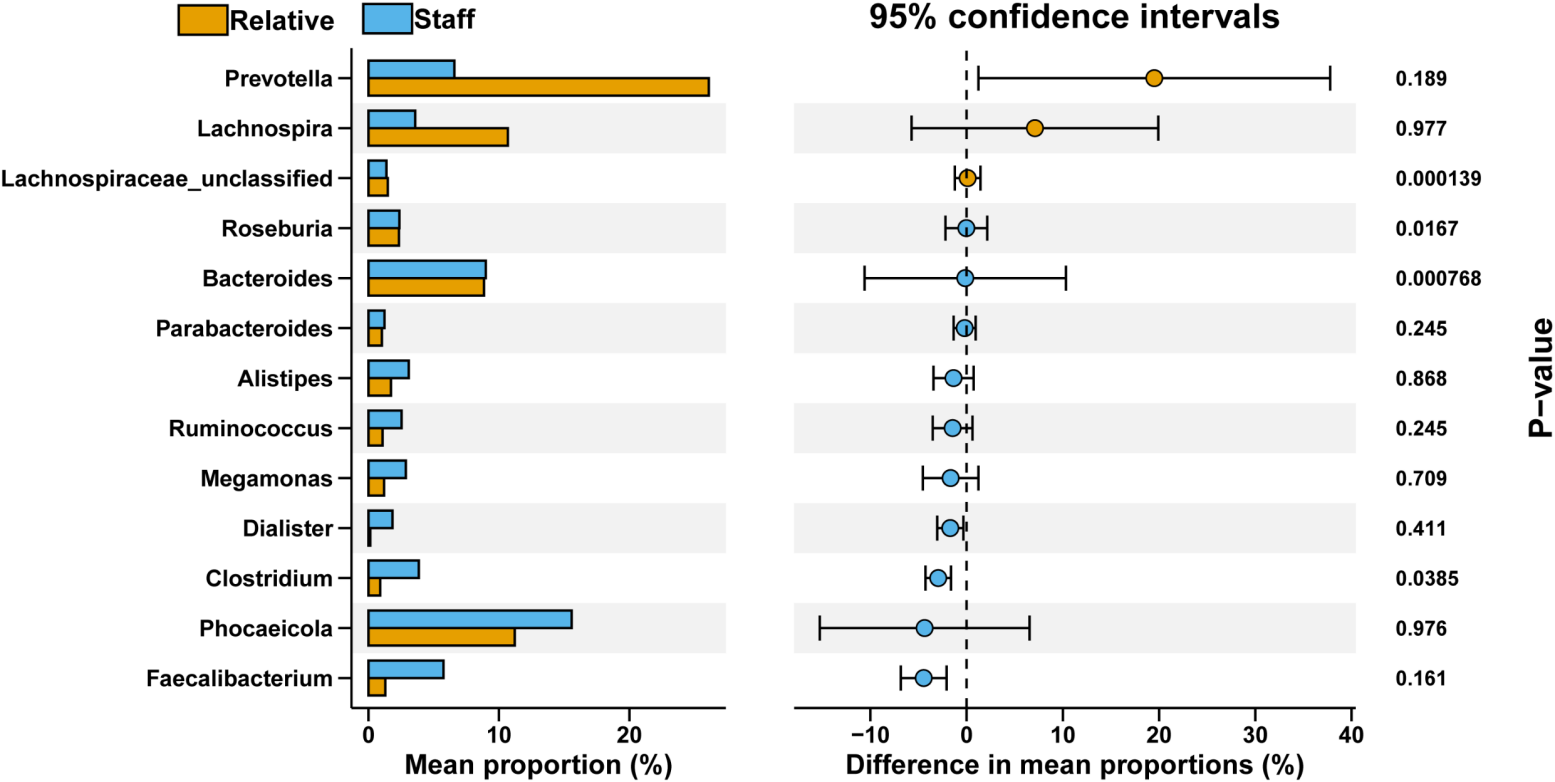
Comparative analysis of bacterial genus proportions between Ward.Relative and Ward.Staff groups. The bar graph illustrates the mean percentage composition of each bacterial genus, with the Relative group represented in orange and the Staff group in blue. The right panel displays the 95% confidence intervals for differences in mean proportions, along with P-values denoting the statistical significance of these differences. Notably, *Lachnospiraceae_unclassified* and *Bacteroides* demonstrate significant variation between groups (P <0.05), highlighting a potentially meaningful distinction in microbial composition.

Furthermore, in the comparison between the Ward.Staff and Ward.Relative groups, we observed a decrease in *Prevotella* and *Lachnospira* in the Ward.Staff group. Additionally, *Lachnospiraceae_unclassified*, *Roseburia*, and *Bacteroides* exhibited significant differences (P<0.05), with *Lachnospiraceae_unclassified* decreasing and *Roseburia* and *Bacteroides* increasing in the Ward.Staff group (**Figure 7**). These findings suggest that changes in the abundance of microbiota potentially associated with CRC development may occur between the Ward.Staff and Ward.Relative groups, indicating that this population may experience a degree of gut microbiota dysbiosis.

### Comprehensive model construction and analysis of POD values

To estimate the probability of disease (POD) for colorectal cancer (CRC) in individuals with CRCR, we developed a comprehensive metagenomic CRC classification model utilizing gut microbiome datasets from nine cohorts spanning diverse global regions. The analysis focused on the top differentially abundant species as optimal markers across these cohorts. A total of 85 differentially abundant species were identified, representing the most effective biomarker panel for distinguishing CRC from Control groups. The differential markers identified within each cohort were encompassed within these 85 species, demonstrating substantial overlap across cohorts.

**Figure 8:**
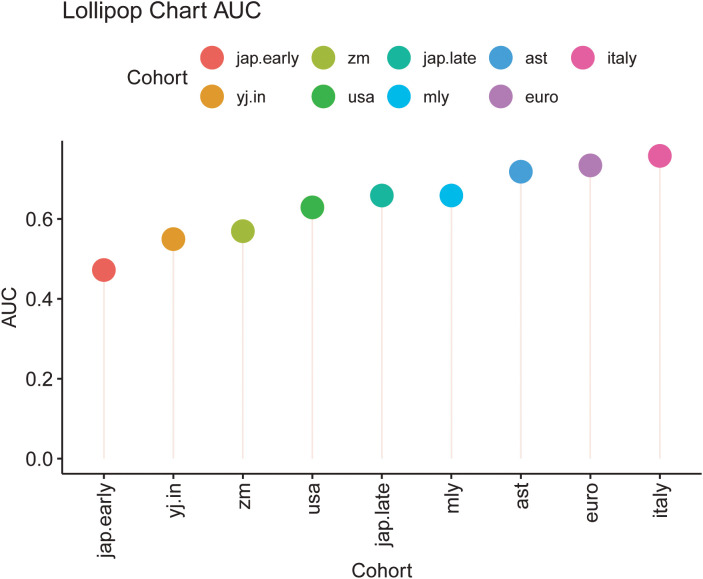
Lollipop chart depicting the area under the curve (AUC) values for different cohorts. Each lollipop indicates the AUC value for a specific cohort, with the lollipop’s length corresponding to the AUC magnitude. Cohorts are color-coded as follows: jap.early (red), zm (green), jap.late (teal), ast (blue), italy (pink), yj.in (orange), usa (dark green), mly (light blue), and euro (purple). This chart facilitates a comparative evaluation of model performance across the cohorts, highlighting variations in AUC values among diverse groups.

**Figure 9:**
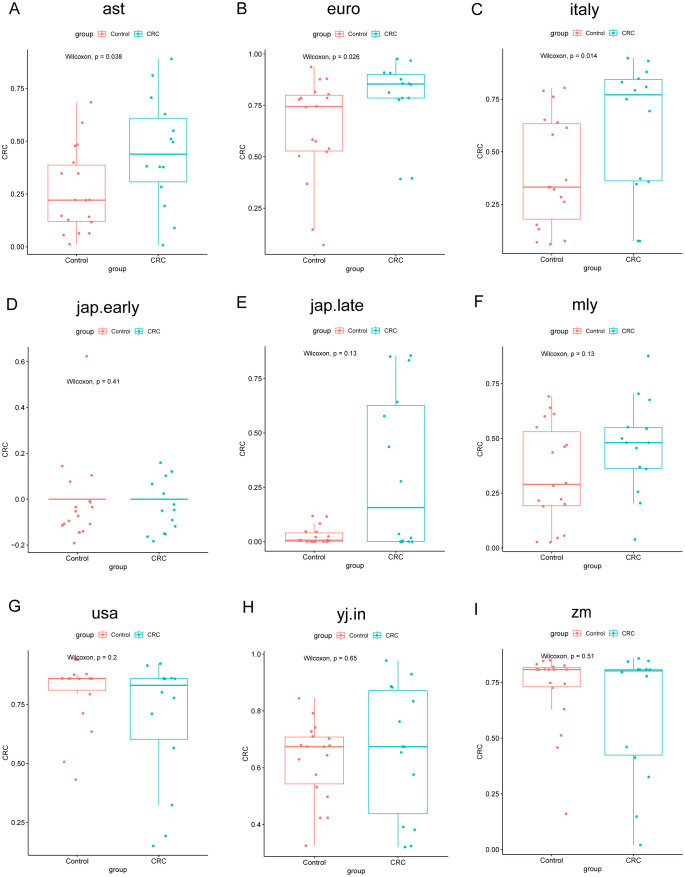
Comparison of POD values across different groups in the Zhengzhou2022 cohort using nine random forest models. The models assess the probability of developing colorectal cancer (CRC). Each subplot **(A–I)** corresponds to one of the random forest models. The x-axis indicates the group, whereas the y-axis represents the POD value. In these boxplots, Control represents healthy controls, and CRC represents CRC patients. P-values were calculated using the Wilcoxon rank-sum test.

To evaluate model performance, data from the Zhengzhou 2022 cohort were used as an independent external dataset for model testing. The area under the curve (AUC) values for the models corresponding to different cohorts were as follows: ast (0.7183), euro (0.7341), italy (0.7579), jap.early (0.4722), jap.late (0.6587), myl (0.6587), usa (0.6290), yj.in (0.5496), and zm (0.5694) (**Supplementary Figures S5A–I**). These results highlight the model’s utility and variability across diverse cohorts. The chart indicates that the classification performance is relatively better for the Italy, Euro, AST, and MYL models, as reflected by their moderately higher AUC values (**Figure 8**). Similarly, the construction of Random Forest Classifier (RFC) models further emphasized the effectiveness of fecal microbiota in distinguishing CRC from control groups. Using these models, the Probability of Disease (POD) was calculated for each individual in the Zhengzhou 2022 cohort. In the AST, Euro, Italy, and MYL models, the CRC group demonstrated significantly higher POD values compared with the control group (**Figures 9A–I**). Overall, the Italy model showed the best ability to distinguish CRC from Control. Therefore, we consider the Italy model to be the most suitable for our study.

Next, we calculated the POD values for the samples from the Zhengzhou 2018 and Zhengzhou 2023 cohorts for validation purposes. AUC values are displayed in (**Supplementary Figures S6**, **S7A–I**). From the AUC values, we can observe that the Random Forest model disease classifier performed significantly worse in the Zhengzhou 2018 cohort compared with the Zhengzhou 2023 cohort. This may be because the two groups in the Zhengzhou 2018 cohort were non-disease groups to begin with, making it difficult for the model to distinctly differentiate between them. In contrast, in the Zhengzhou 2023 cohort, the model was used to distinguish between the disease group (Ward.CRC) and the non-disease groups (Ward.Relative and Ward.Staff), which resulted in better classification performance.

**Figure 10:**
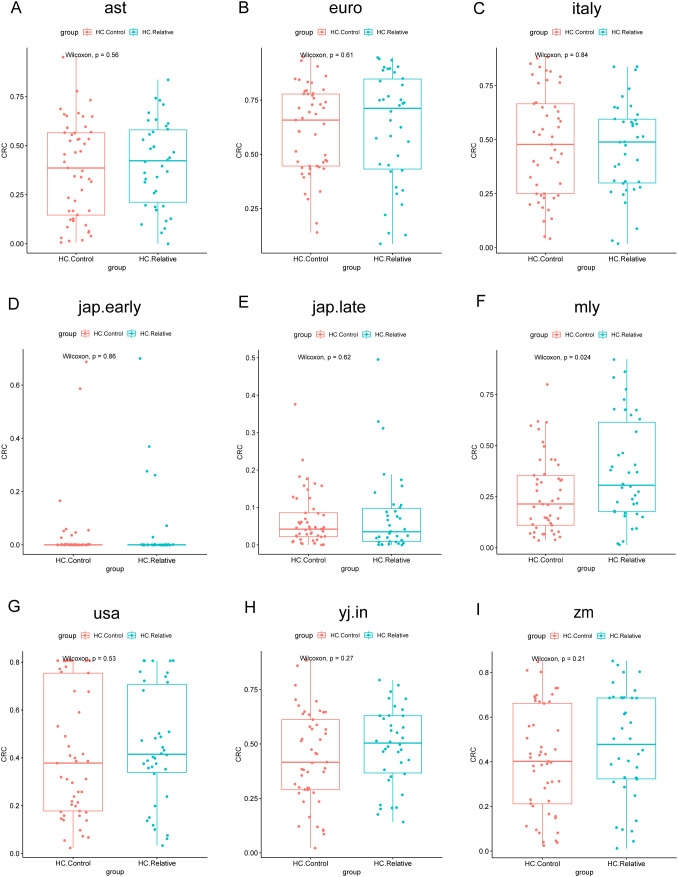
Comparison of POD values across different groups in zhengzhou2018 cohort using nine random forest models. The models assess the probability of developing colorectal cancer (CRC). Each subplot **(A–I)** corresponds to one of the random forest models. The x-axis indicates the group, whereas the y-axis represents the POD value. In these boxplots, HC.Control represents healthy controls, and HC.Relative represents healthy relatives of CRC patients. P-values were calculated using the Wilcoxon rank-sum test.

In the Zhengzhou 2018 cohort, except for the jap.early and jap.late models, the other seven models showed that the POD values for the HC.Relative group were higher than those for the HC.Control group (**Figures 10A–I**), despite no significant statistical differences between the two groups.

**Figure 11:**
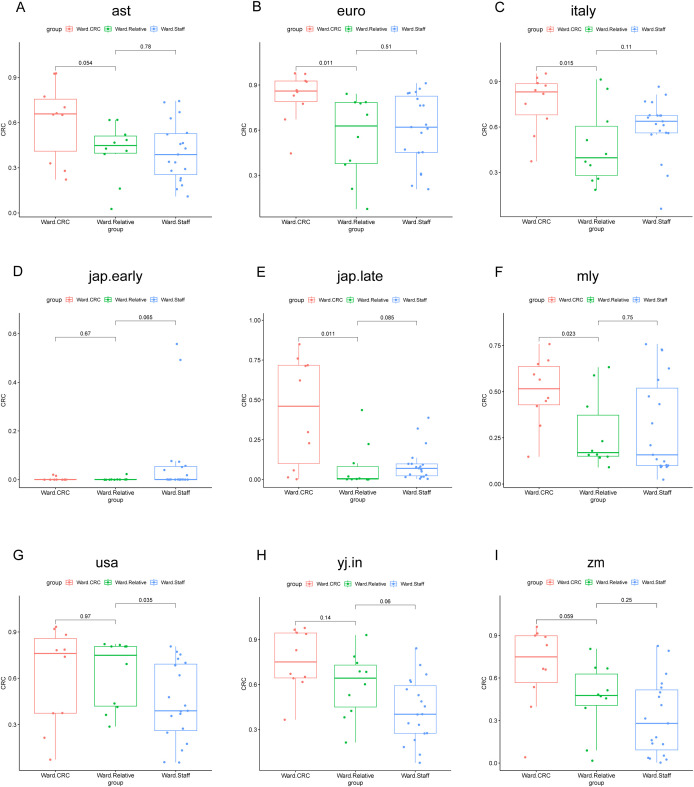
POD value comparisons across different groups and models in the Zhengzhou2023 cohort. This figure illustrates the comparison of POD values among three groups (Ward CRC, Ward Relative, and Ward Staff) using nine different random forest models. Each subplot **(A–I)** corresponds to a specific model: **(A)** ast, **(B)** euro, **(C)** italy, **(D)** jap.early, **(E)** jap.late, **(F)** mly, **(G)** usa, **(H)** yj.in, and **(I)** zm. The x-axis represents the group classification (Ward CRC, Ward Relative, and Ward Staff), whereas the y-axis indicates the POD values. The boxplots depict the distribution of POD values within each group. P-values, derived from the Wilcoxon rank-sum test, are shown for pairwise comparisons between the groups. Significant differences are indicated where applicable.

In the Zhengzhou 2023 cohort, the POD values for the Ward.CRC group were markedly higher than those for the Ward.Relative and Ward.Staff groups. Furthermore, we observed that the comparison of POD values between the Ward.Relative and Ward.Staff groups showed varying results across different models, which may be attributed to the heterogeneity of the population and the impact of sample size. Based on the AUC values mentioned earlier, we continue to focus on the results from the Italy model. We found that the POD values for the Ward.Staff group were higher than those for the Ward.Relative group (**Figures 11A–I**). The gut microbiota of healthcare staff, who have prolonged exposure to CRC patients, may be influenced to some extent. These findings suggest that the POD values for first- and second-degree relatives of colorectal cancer patients, as well as individuals who have lived in the same environment as CRC patients for an extended period, are elevated compared with individuals without a family history of colorectal cancer.

### Correlation between red meat intake and microbial features

In the 2018 Zhengzhou cohort, we observed a significant difference in red meat intake (red meat >150 g/w) between HC.Relative and HC.Control groups. A greater number of individuals in the patient relative group consumed more than 150 g of red meat per week compared with the healthy control group. Red meat has been established as a risk factor for CRC. Therefore, we performed a correlation analysis between red meat intake and microbial features. The microbial species significantly correlated with red meat intake are listed in (**Supplementary Table S1**). Notably, several bacteria potentially associated with CRC development, such as *Ruminococcus_sp_AF41_9, Prevotella_copri_clade_E*, and *Enterococcus_faecalis*, were enriched in the CRC relative group. Additionally, there was a reduction in some beneficial bacteria, including *Eubacterium_rectale*, *Gemmiger_formicilis*, *Lachnospiraceae_bacterium_OF09_6*, and *Phocaeicola_plebeius*. These bacteria are typically involved in the production of butyrate and short-chain fatty acids, which play a protective role in the gut.

### Follow-up results of the zhengzhou 2018 cohort

We performed a 3–4-year follow-up with each participant from zhengzhou2018. We based on the following question to make a follow-up: for the HC.Rrelative group—the diagnosis age of CRC patients, the survival state of the CRC patients now, and cohabitation condition with CRC patients; for both HC.Rrelative group and HC.Control group—adherence to colonoscopy screening and colonoscopy results. Totally, we obtained the follow-up information from 23 participants in the HC.Rrelative group and 26 participants in the HC.Control group. Participants who were unable to be contacted due to incorrect phone numbers, refusal to respond to our questions, etc.

HC.Rrelative participants demonstrated more adherence to colonoscopy (11/23), whereas only 8 out of 26 control participants underwent colonoscopy. The self-reported colonoscopy results indicated that HC.Rrelative participants were vulnerable to suffering polyps, although it was no statistical difference: 9 out of 11 (81.82%) HC.Rrelative participants with self-reported polyps versus 4 out of 8 (50.00%) HC.Control participants. All participants reported that their polyps were benign (**Supplementary Figure S8**). There were 2 out of 23 HC.Rrelative participants who were reticent to provide information about their CRC relatives. Three CRC patients were diagnosed under the age of 50, and nine CRCR patients were non-survivors (**Table 3**).

**Table 3:** Colonoscopy follow-up results of the zhengzhou2018 cohort.

Total follow-up HC.Relative participants		23
HC.Relative diagnosis age	<50	3
	≥50	18
HC.Relative survival state	Survival	12
	Non-survival	9
Cohabitation	Yes	7
	No	14

## Discussion

CRC patients’ relatives were proven at a high risk to suffer from CRC. However, there have been no studies yet to identify gut microbiota characteristics of the unaffected relatives. Here, we first compared the gut microbiome composition of unaffected relatives of colorectal cancer (CRC) patients and healthy individuals without a family history of CRC. We observed an increased abundance of *Parabacteroides*, *Proteobacteria_unclassified*, and *Bacteroides* in the CRC relatives (CRCR group), alongside a reduction in beneficial bacteria such as *Lachnospiraceae_unclassified* and *Megamonas*. The Probability of Disease (POD) discrimination model, validated with external datasets, showed higher POD values in CRCR participants compared with healthy controls, indicating a higher likelihood of CRC in this group. Additionally, to assess the impact of both genetic and environmental factors, we included staff members working in the gastroenterology ward. The results revealed that the POD values for *Ward.Staff* were higher than those for *Ward.Relative*, suggesting that environmental exposure to CRC patients might contribute to shifts in gut microbiota that are also reflected in higher POD values. Furthermore, follow-up colonoscopy data from the Zhengzhou 2018 cohort showed that unaffected relatives of CRC patients were more susceptible to developing polyps than healthy controls, although the difference was not statistically significant.

CRC is the third mortality and the fourth most common cancer (Rawla et al., 2019). CRC carcinogenesis is a result of comprehensive effects of genetics and environmental exposures, of which genetics contributes 35%–40% parts (Keum and Giovannucci, 2019). Hereditary CRC counts for 7%–10% of the total CRC cases. Hereditary nonpolyposis colorectal cancer, known as HNPCC or Lynch syndrome, is a frequent hereditary CRC and an autosomal dominant inheritance, which usually carries gene mutations as MLH1, MSH2, MSH6, EpCAM, and PMS2 (Bonadona et al., 2011; Tutlewska et al., 2013). Genetic information alteration also exists in sporadic CRC, including epigenetic alterations, activation of oncogenes KRAS and PIK3CA, and mutation in tumor-suppressor genes such as TP53 and APC (Carethers and Jung, 2015; Sobhani et al., 2019). Gene alteration offers individual susceptibility, whereas environmental factors mainly promote tumorigenesis (Kune, 2010). Unhealthy lifestyles, including loss of physical activity, fat, excessive red meat consumption, and alcohol and smoking exposures, have been linked to risk factors (Zisman et al., 2006; Gingras and Béliveau, 2011).

Gut microbiome transmission between humans exists in a variety of ways (Browne et al., 2017). Whole-metagenome shotgun sequencing of intestinal microbiota confirmed the coherence of species within families. Kinship and cohabitation both contributed to gut microbiome transmission (Valles-Colomer et al., 2022). For the rodent model, gut microbiome transmission can result in similar disease phenotypes. Wild-type mice cohabited with inflammasome-deficiency mice resulting in gut microbiome alteration of wild-type mice, which resembled those of inflammasome-deficient mice. In addition, co-housing resulted in liver inflammation of wild-type mice, indicating that cohabitation can lead to microbiota-related diseases through the transmission of intestinal microbes (Henao-Mejia et al., 2012). This suggests that long-term exposure to the same living environment as colorectal cancer patients may contribute to certain microbial transmission.

We identified alterations in microbial genera within the CRCR groups, correlating with changes observed in colorectal cancer (CRC) patients. Notably, the genera *Clostridium, Bacteroides, Parabacteroides, Proteobacteria_unclassified*, and *Coprococcus* were significantly increased in unaffected relatives (Liu et al., 2016; Patterson et al., 2017; Chen et al., 2018). Conversely, species such as *Lachnospiraceae_unclassified* and *Megamonas* exhibited notable reductions. These genera are intricately linked to both gut health and disease states (Gupta et al., 2019; Yang et al., 2021). Former study approved that *C. difficile* could promote CRC tumorigenesis by activating the Wnt pathway through its virulence factor TcdB (Drewes et al., 2022). As reported, certain *Bacteroides* species, such as *B. fragilis*, can contribute to CRC through mechanisms like toxin production and immune system modulation. *Bacteroides fragilis*, specifically the enterotoxigenic strain (ETBF), has been shown to produce toxins that may promote chronic inflammation, oxidative DNA damage, and epithelial barrier disruption, all of which are conducive to carcinogenesis. Research has identified higher frequencies of *B. fragilis* in CRC patients compared with controls, indicating its potential role in tumor formation (Boleij et al., 2015). Furthermore, *B. fragilis* is often found in higher concentrations in the stool samples of CRC patients, and its presence correlates with more advanced stages of the disease (Haghi et al., 2019)​.

Similarly, in the zhengzhou2018 cohort, the HC.Relative group showed elevated levels of *Parabacteroides* compared with the HC.Control group. Previous studies have consistently reported a notable increase in *Parabacteroides* abundance in CRC patients, which may be attributed to its pro-inflammatory properties. *Parabacteroides* produces metabolites such as lipopolysaccharides (LPS), which are known to induce inflammatory responses. Chronic inflammation, in turn, is closely associated with the development of CRC. Additionally, secondary bile acids such as deoxycholic acid, also produced by certain gut microbes, can damage intestinal epithelial cells or promote tumor growth, thereby facilitating cancer progression (Yu et al., 2017; Zackular et al., 2013; Zhu et al., 2024). In addition, *Faecalibacterium* enriched in HC.Relative group. This genus is well known for its probiotic properties and its role in maintaining gut health. *Bifidobacterium* species can help balance the gut microbiota, improve digestion, and enhance immune function. According to colonization resistance, the growth of probiotics inhibits the overgrowth of bacteria, which were normal in low abundance to maintain the healthy mammalian intestinal tract (Bermudez-Brito et al., 2012; Lawley and Walker, 2013; Vazquez-Gutierrez et al., 2016). Therefore, the identification and characterization of CRC-associated bacteria are of critical importance.

The POD index model was developed, suggesting a higher probability of CRC in unaffected relatives, although this observation did not achieve statistical significance. Genetic predisposition may play a role in how an individual’s microbiome responds to or coexists with other microbes, influencing factors like inflammation, immune response, and metabolic health. Such genetic and environmental interactions can also impact disease risk, including the development of conditions such as colorectal cancer (Zamani et al., 2019).

Due to the heterogeneity of the population and the impact of sample size, we calculated the POD values for Zhengzhou2018 and Zhengzhou2023 using different disease models, which yielded varying results. Based on the most suitable model (Italy), it was revealed that the POD values for the Ward.Staff group were higher than those for the Ward.Relative group. The Ward.Staff group consisted of individuals who work daily in close proximity to colorectal cancer patients. In contrast, the Ward.Relative group primarily comprised first-degree relatives, typically sons or daughters, who may not have lived with the patients for extended periods due to work or study commitments. As a result, these relatives are less likely to have been in constant contact with the patients. In contrast, the ward staff, who spend prolonged time in the same environment, are likely to experience microbial transmission due to shared exposure, which could contribute to the similarity in gut microbiota. The analysis of the intestinal microbiome in CRCR individuals or environmental cohabitants suggested that the gut microbiome can be transmitted between relatives and those living in the same environment. However, whether the transmission of gut microbiome among humans causes disease-related phenotype alteration is an ambitious topic that needs to be confirmed in a large number of population and animal studies.

Colonoscopy follow-up results suggested a higher incidence of polyps in the HC.Relative group, whereas the results were not significant, probably due to the limited sample size. The HC.Relative group showed a stronger adherence to colonoscopy than control group. However, the adherence still did not meet the recommended criteria. For the background population, the first colonoscopy at the age of 50 is recommended (Rex et al., 2017). When one’s FDRs are diagnosed with sporadic CRC before 60 years old, they should undergo colonoscopy every 5 years at 40 years old, or 10 years before the diagnostic age of the FDRs (Rex et al., 2017). As for a person with an SDR diagnosed with CRC, screening the colonoscopy at age 50 years is suggested (Wilkinson et al., 2019).

In terms of dietary habits, previous studies have shown that red meat consumption is a risk factor for CRC (Oostindjer et al., 2014). This may be partially explained by the adverse effects of certain processing methods on gut health, such as increased inflammation. Additionally, excessive red meat intake may reduce the consumption of other nutrient-dense foods, thereby raising the risk of disease (Schwingshackl et al., 2018; Song et al., 2020). Interestingly, our study revealed that in zhengzhou2018 cohort, the relatives of CRC patients reported higher red meat consumption compared with healthy individuals without a family history of CRC. However, this difference was not observed for white meat intake. In the Zhengzhou 2023 cohort, no significant differences were observed in the consumption of red or white meat among CRC patients, their relatives, and ward staff. Some bacteria potentially associated with CRC development were enriched in the group with high red meat intake, whereas beneficial bacteria were significantly reduced. This reflects that excessive red meat consumption alters the composition of the gut microbiota and may be a contributing factor to disease development.

The link between dietary patterns and CRC has been well established, and maintaining gut health requires a comprehensive approach (Liang et al., 2023). Although our analysis did not find significant differences in whole grain or probiotic intake across groups, we believe that probiotics still play a beneficial role in supporting gut health. The gut microbiota is highly dynamic and complex, but previous studies have demonstrated that supplementation with a mixture of 17 probiotic strains can restore normal gut immune function (Tripathy et al., 2021). Thus, dietary patterns that support gut health may contribute to shaping a healthier gut microbiota and reducing the risk of CRC.

The limitations of this study include the small sample size of unaffected relatives. Additionally, non-related family members of CRC patients (such as spouses) were not included in this research. Incorporating non-related family members alongside matched first-degree relatives may enhance the robustness of our findings. Finally, the Ward.Relative participants paired with Ward.CRC have not yet reached the follow-up period, leaving us unable to predict whether the probability of developing colorectal cancer in these participants over the next 5 years will exceed that of healthy individuals without a family history of CRC.

## Conclusion

Overall, our findings indicate microbial dysbiosis in unaffected relatives, suggesting potential early microbial alterations associated with CRC risk. However, these associations do not necessarily imply causation or direct microbial transmission between affected individuals and their relatives. Instead, they may reflect shared environmental factors, dietary patterns, or genetic predispositions that influence gut microbiome composition and, consequently, CRC susceptibility. Further research is needed to disentangle the complex interplay between genetic factors, environmental influences, and gut microbiome dynamics in CRC pathogenesis.

## Data Availability

The datasets generated and analyzed during the current study are available in the CNGB Sequence Archive (CNSA) of the China National GenBank Database (CNGBdb) at https://db.cngb.org/, reference number CNP0006624.
