# Supplementary material for: Gut microbiome differences and disease risk in colorectal cancer relatives and healthy individuals
Source: Front Cell Infect Microbiol. 2025 Mar 24;15:1573216. doi: 10.3389/fcimb.2025.1573216 (PMC11973321; doi:10.3389/fcimb.2025.1573216)
Supplement: Supplementary file 1 [file DataSheet1.docx]

**Supplementary Figures and tables**

**
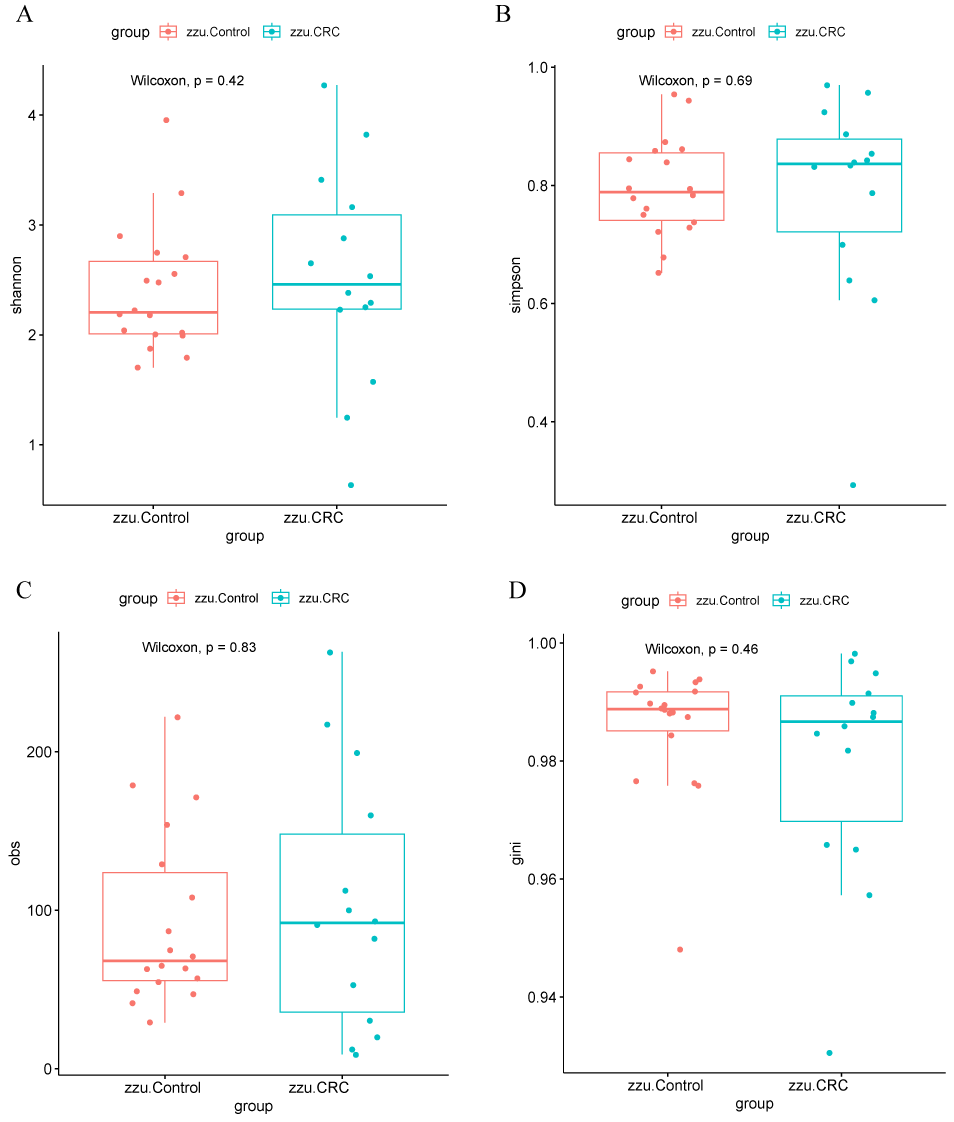
**

**Fig. S1: Alpha Diversity Metrics in zzu.Control and zzu.CRC Groups**

(A) Shannon diversity index comparison between zzu.Control group and zzu.CRC group, showing no significant difference (Wilcoxon test, p = 0.42). (B) Simpson diversity index comparison, with no significant difference observed (Wilcoxon test, p = 0.69). (C) Observed species (obs) metric comparison, with no significant difference between groups (Wilcoxon test, p = 0.83). (D) Gini coefficient comparison, also with no significant difference (Wilcoxon test, p = 0.46). These findings indicate no significant differences in alpha diversity metrics between Zou's control and CRC groups.

**
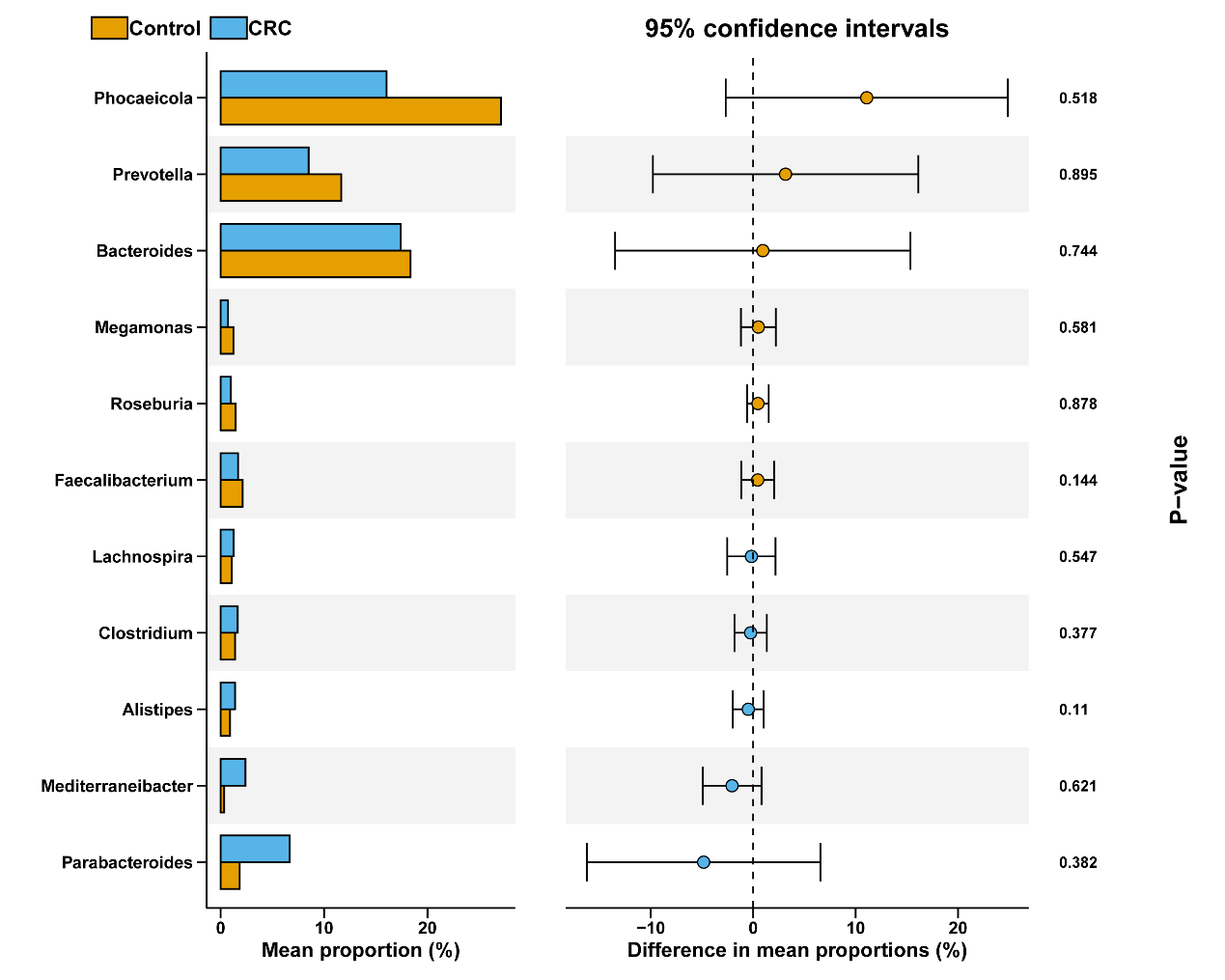
**

**Fig. S2:Comparison of gut microbiota between zzu.Control and zzu.CRC groups**. The bar plot on the left depicts the mean proportion of bacterial taxa, while the right panel illustrates the difference in mean proportions between the two groups, with 95% confidence intervals. P-values are provided for significance testing of microbial differences. *Parabacteroides*, *Prevotella* and *Phocaeicola* show prominent differences in abundance, however, these differences are not statistically significant.


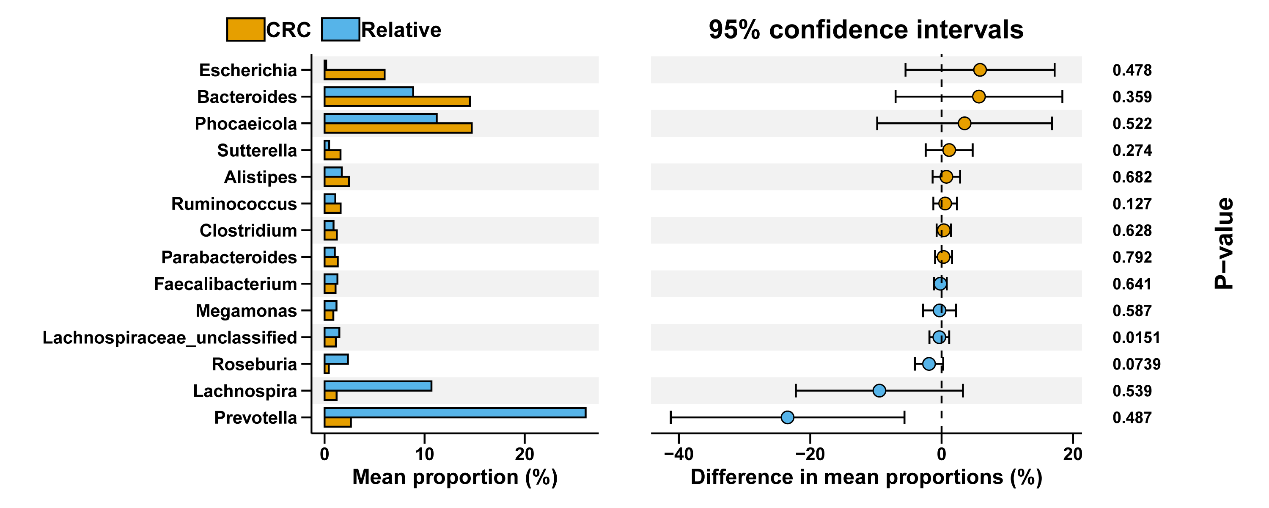


**Fig. S3: Comparative analysis of microbial genus proportions between Ward.CRC and Ward.Relative groups.** The bar chart depicts the mean percentage composition of bacterial genera in CRC (orange) and Relative (blue) groups. The left x-axis indicates the mean proportions of each genus, while the right x-axis highlights the mean differences between groups, accompanied by 95% confidence intervals. Statistical significance of group differences is denoted by P-values, with *Lachnospiraceae_unclassified* showing a significant difference (P = 0.0151), suggesting a notable divergence in microbial composition.

**
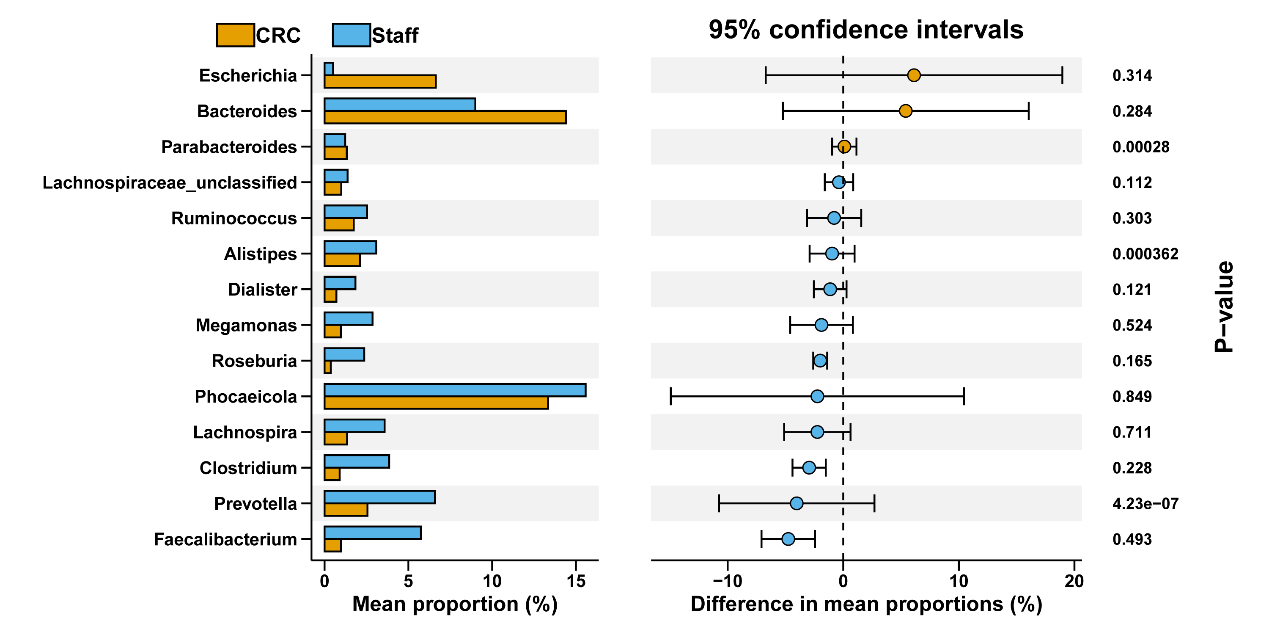
**

**Fig****. S4 Microbiome composition comparison between Ward.CRC and Ward.Staff groups.** The bar plot on the left shows the mean proportion of bacterial taxa, and the right panel presents the difference in mean proportions with 95% confidence intervals. Significant microbial differences between the groups, such as in *Parabacteroides, Alistipes* and *Prevotella* are indicated, along with p-values for significance levels.


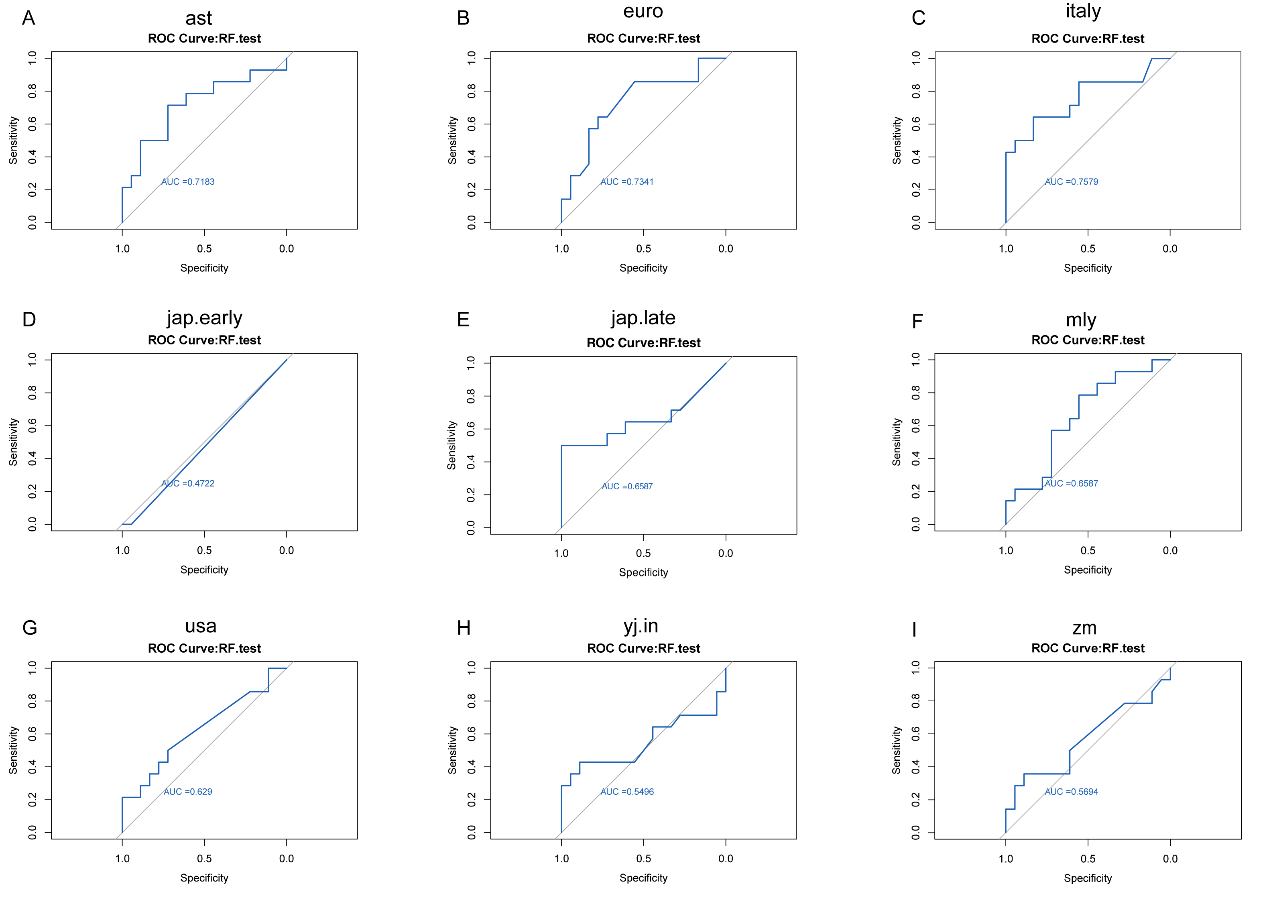


**Fig. S5 AUC values of zhengzhou2023 cohort.** Receiver Operating Characteristic (ROC) curves for nine random forest models evaluated with the 2022 cohort data. Panels A to I correspond to the models: *ast* (A), *euro* (B), *italy* (C), *jap.early* (D), *jap.late* (E), *myl* (F), *usa* (G), *yj.in* (H), and *zm* (I). The Area Under the Curve (AUC) values, ranging from 0.4722 to 0.7579, are annotated on each plot, representing each model's predictive accuracy.

**
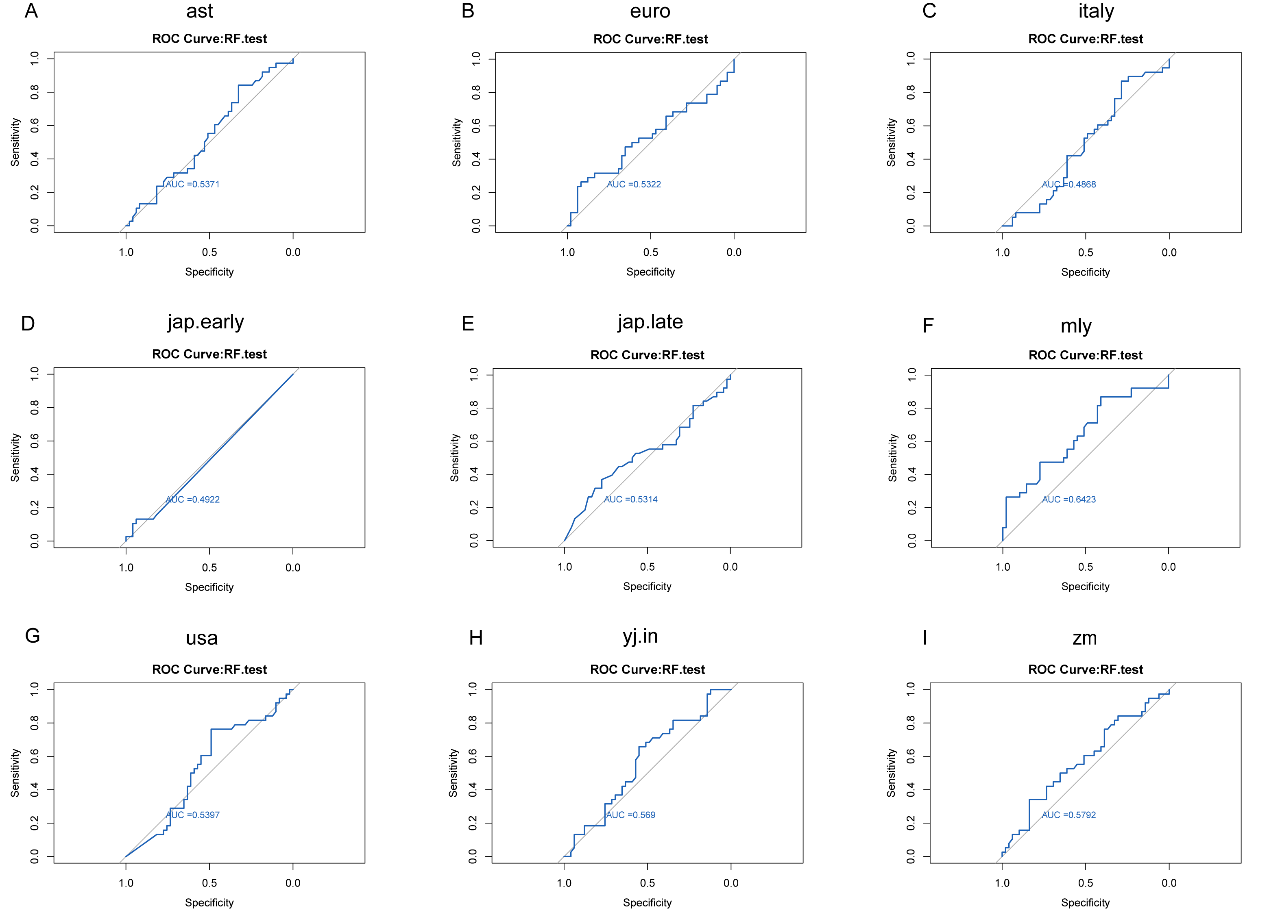
 Fig. S6 AUC values of zhengzhou 2018 cohort.** Receiver Operating Characteristic (ROC) curves for nine random forest models applied to the 2018 cohort data. A-I represent the models: ast (A), euro (B), italy (C), jap.early (D), jap.late (E), myl (F), usa (G), yj.in (H), and zm (I). Each curve illustrates the model's capacity to distinguish between classes, with the Area Under the Curve (AUC) values displayed on the respective plots. AUC values range from 0.4922 to 0.6423, reflecting diverse predictive accuracies across models. These metrics underscore the models' diagnostic and prognostic potential for the 2018 cohort.


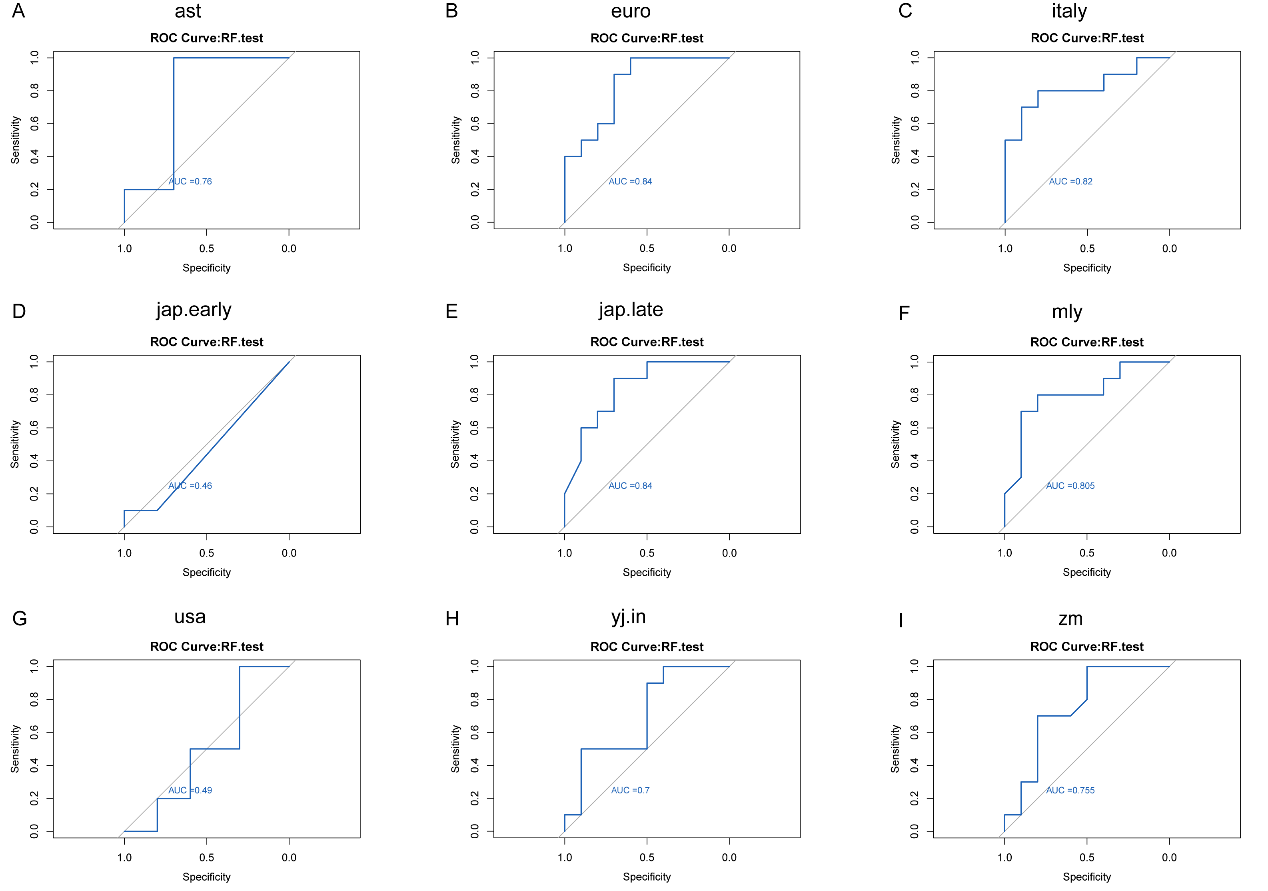


**Fig.S7 AUC values of zhengzhou2023 cohort.** This figure displays the Receiver Operating Characteristic (ROC) curves for nine random forest models, each evaluated using the 2023 cohort data. The models, labeled as ast (A), euro (B), italy (C), jap.early (D), jap.late (E), myl (F), usa (G), yj.in (H), and zm (I), are assessed based on their ability to discriminate between classes within the cohort. The AUC values, which range from 0.48 to 0.84, are indicated on each plot, providing a quantitative measure of each model's predictive accuracy. The ROC curves and AUC values are essential for understanding the models' performance and for selecting the most effective model for further analysis within the 2023 cohort.


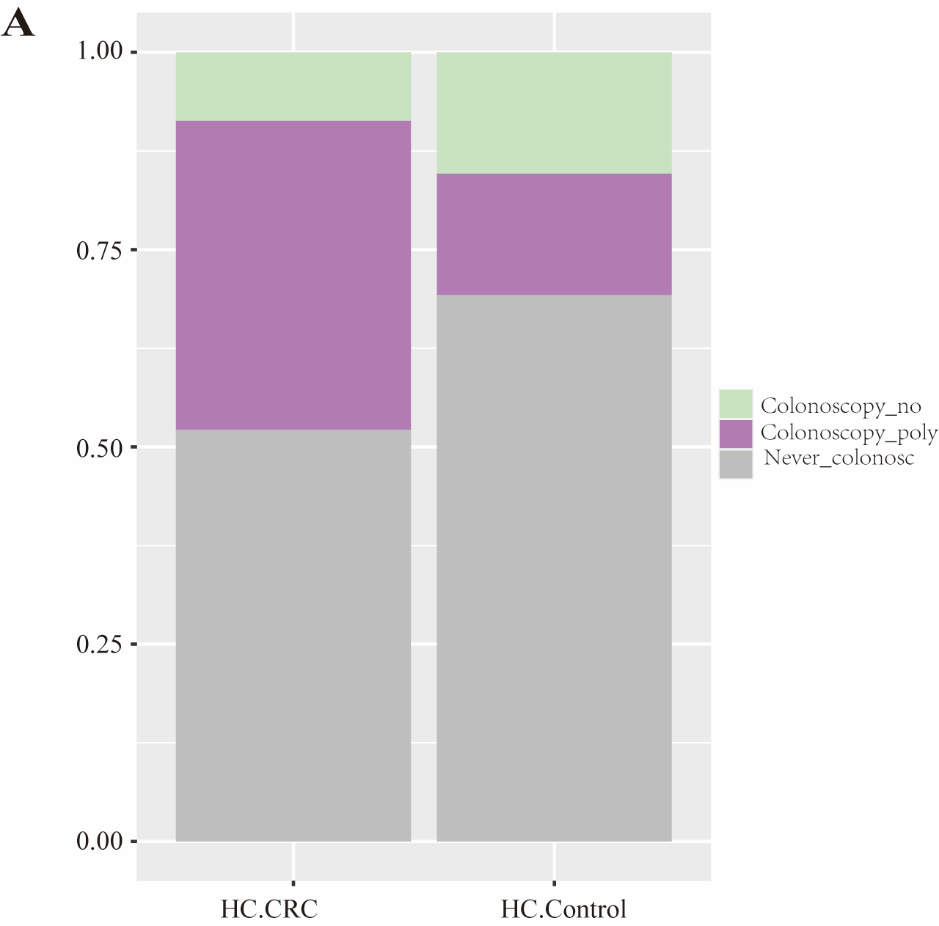


**Fig. S8: Stacked bar chart comparing the proportions of colonoscopy outcomes between the HC.CRC and HC.Control groups.** Outcomes are categorized as Colonoscopy_no (light green), Colonoscopy_poly (purple), and Never_colonosc (gray). Each bar represents the overall distribution of these categories within each group, with bar height indicating the percentage contribution of each category. HC.CRC participants showed greater adherence to colonoscopy (11/23), compared to only 8 out of 26 participants in the HC.Control group undergoing colonoscopy.

**Table S1 Correlation of the relationship between microbial features and red meat intake in 2018 cohort**

| **Feature(species)** | **metadata** | **rankRho** | **rankPvalue** |
| --- | --- | --- | --- |
| Eubacterium_rectale | Red_meat_150g_w | -0.25673 | 0.033222 |
| Gemmiger_formicilis | Red_meat_150g_w | -0.28278 | 0.018556 |
| Prevotella_copri_clade_A | Red_meat_150g_w | -0.0806 | 0.510306 |
| Phocaeicola_plebeius | Red_meat_150g_w | -0.25715 | 0.032919 |
| Bacteroides_finegoldii | Red_meat_150g_w | -0.24136 | 0.045729 |
| Coprococcus_eutactus | Red_meat_150g_w | -0.23761 | 0.049311 |
| Clostridiaceae_unclassified_SGB4771 | Red_meat_150g_w | -0.25532 | 0.03423 |
| Clostridium_butyricum | Red_meat_150g_w | 0.284095 | 0.017995 |
| Ruminococcus_sp_AF41_9 | Red_meat_150g_w | -0.26218 | 0.029537 |
| Faecalimonas_umbilicata | Red_meat_150g_w | 0.267269 | 0.026412 |
| Tyzzerella_nexilis | Red_meat_150g_w | 0.222765 | 0.065796 |
| Candidatus_Nanosynsacchari_sp_TM7_ANC_38_39_G1_1 | Red_meat_150g_w | 0.252804 | 0.036103 |
| Lachnospiraceae_bacterium_OF09_6 | Red_meat_150g_w | -0.34027 | 0.004226 |
| Negativibacillus_massiliensis | Red_meat_150g_w | 0.275237 | 0.022084 |
| Candidatus_Avimonas_narfia | Red_meat_150g_w | 0.208981 | 0.084834 |
| Streptococcus_anginosus | Red_meat_150g_w | 0.295073 | 0.013841 |
| Streptococcus_sanguinis | Red_meat_150g_w | 0.295073 | 0.013841 |
| TM7_phylum_sp_oral_taxon_348 | Red_meat_150g_w | 0.267367 | 0.026355 |
| Streptococcus_oralis | Red_meat_150g_w | 0.267367 | 0.026355 |
| Prevotella_copri_clade_E | Red_meat_150g_w | 0.267367 | 0.026355 |
| Megasphaera_micronuciformis | Red_meat_150g_w | 0.253368 | 0.035677 |
| Clostridiales_bacterium_NSJ_40 | Red_meat_150g_w | 0.250318 | 0.038036 |
| Acidaminococcus_provencensis | Red_meat_150g_w | 0.250318 | 0.038036 |
| Eggerthella_sinensis | Red_meat_150g_w | 0.250318 | 0.038036 |
| Enterococcus_faecalis | Red_meat_150g_w | 0.2745 | 0.022457 |
| Anaerostipes_hominis | Red_meat_150g_w | 0.237376 | 0.049537 |
| Senegalimassilia_SGB14826 | Red_meat_150g_w | 0.237376 | 0.049537 |
| Blautia_SGB6487 | Red_meat_150g_w | 0.237376 | 0.049537 |

Ranking of correlation coefficients and significance testing. p<0.05.
